# Latitudinal gradients in the phylogenetic assembly of angiosperms in Asia during the Holocene

**DOI:** 10.1038/s41598-024-67650-1

**Published:** 2024-08-02

**Authors:** Kuber P. Bhatta, Ondřej Mottl, Vivian A. Felde, John-Arvid Grytnes, Triin Reitalu, Hilary H. Birks, H. John B. Birks, Ole R. Vetaas

**Affiliations:** 1 Department of Biological Sciences, University of Bergen, PO Box 7803, 5020 Bergen, Norway; 2 Bjerknes Centre for Climate Research, 5020 Bergen, Norway; 3 Center for Theoretical Study, Charles University, Jilská 1, 11000 Prague, Czech Republic; 4 Department of Botany, Faculty of Science, Charles University, Benátská 2, 12801 Prague, Czech Republic; 5 Institute of Ecology and Earth Sciences, University of Tartu, J. Liivi tn 2, 50409 Tartu, Estonia; 6 Institute of Geology, Tallinn University of Technology, Ehitajate tee 5, 19086 Tallinn, Estonia; 7 Environmental Change Research Centre, University College London, Gower Street, London, WC1 6BT UK; 8 Department of Geography, University of Bergen, PO Box 7802, 5020 Bergen, Norway

**Keywords:** Ecology, Biogeography, Palaeoecology

## Abstract

Spatio-temporal assessment of phylogenetic diversity gradients during the Holocene (past 12,000 years) provides an opportunity for a deeper understanding of the dynamics of species co-occurrence patterns under environmental fluctuations. Using two robust metrics of phylogenetic dispersion (PD) and 99 fossil pollen sequences containing 6557 samples/assemblages, we analyse spatio-temporal variation in PD of angiosperms and its relationship with Holocene climate in central Asia. Overall, PD throughout the Holocene decreases linearly with increasing latitude, except for a rise in mean nearest taxon distance from ca. 25 to 35° N. This indicates that phylogenetically divergent taxa decrease progressively with increasing latitude, leaving more phylogenetically closely related taxa in the assemblages, thereby increasing phylogenetic relatedness among the co-occurring taxa. The latitudinal gradient of PD has not been consistent during the Holocene, and this temporal variation is concordant with the Holocene climate dynamics. In general, profound temporal changes in the latitudinal PD toward higher latitudes implies that the major environmental changes during the Holocene have driven considerable spatio-temporal changes in the phylogenetic assembly of high-latitude angiosperm assemblages. Our results suggest that environmental filtering and the tendency of taxa and lineages to retain ancestral ecological features and geographic distributions (phylogenetic niche conservatism) are the main mechanisms underlying the phylogenetic assembly of angiosperms along the climate-latitudinal gradient. Ongoing environmental changes may pose future profound phylogenetic changes in high-latitude plant assemblages, which are adapted to harsh environmental conditions, and therefore are phylogenetically less dispersed (more conservative or clustered).

## Introduction

Assembly of plant species is primarily governed by ecological and evolutionary processes along environmental and/or physiographic gradients which create biodiversity gradients. The latitudinal diversity gradient (LDG), a pattern of decreasing biological diversity from the equator to the poles has long been recognised for different groups of organisms across geographic regions^1^. The modern LDG is hypothesised to reflect the long-term effect of eco-evolutionary, biogeographic, and geological factors through evolutionary time^2–4^. This pattern is considered to be stable at time scales of centuries to millennia because the evolutionary and biogeographic processes are assumed to be slow and not detectable at these scales. However, this assumption is not based on empirical evidence of the effect of major environmental variations during past millennia on the dynamics of the present-day LDG for angiosperms. This remains largely unexplored, mostly due to a lack of appropriate data^3^. A test of this hypothesis is critically important for predicting future spatio-temporal variations in angiosperm assemblages under ongoing environmental changes.

Understanding the extent to which the latitudinal gradient in phylogeny of angiosperms has varied over time scales that are too short for observing evolutionary imprints of assemblage dynamics, and to investigate the underlying drivers and mechanisms, requires combining the spatial and temporal patterns of phylogenetic diversity. In this way, we may detect if ecological, evolutionary, or geophysical imprints prevail in explaining the origin of present-day phylogenetic patterns of species co-occurrence along environmental and physiographic gradients, and hence contribute to a robust assessment of the spatio-temporal dynamics of taxa and their assemblages^5–7^. Documenting phylogenetic relationships among co-occurring taxa through time and space and relating the observed variations to potential underlying drivers is also important for gauging conservation efforts required in regions that harbour the most threatened portions of the phylogeny (for example, areas with exceptionally low or high phylogenetic dispersion)^3,8^.

At present, there are very few phylogeny-based assessments of spatio-temporal biodiversity gradients^9–11^, mostly because such assessments require good quality data covering variation in diversity along temporal as well as spatial gradients and appropriate analyses. Temporal information can either be obtained by vegetation re-surveys over decades, or by using palaeo-archives. However, vegetation re-surveys are too scarce, and they cover a rather short temporal span of decades to centuries^12^. Therefore, such studies are mostly useful in assessing short-term and local spatio-temporal dynamics of vegetation assemblages. Temporal information of several centuries to millennia for vegetation over a broader geographic extent can be obtained by using consistently processed and spatially and temporally standardized palaeoecological data, which are archives of past vegetation composition, biodiversity, and environment^13–15^. A fossil pollen record preserves samples of plant taxa that coexisted within a region including their relative abundances, and offers an undisturbed baseline for analysing co-occurrences and mechanisms of biotic assemblages over different spatial and temporal scales^15^. Although taxa recorded in the fossil pollen records are usually not identified to species level and there is no direct 1:1 relationship between a pollen assemblage and a plant assemblage at any taxonomic level, several studies have documented that such data can be used to reconstruct quantitative biodiversity patterns. For such studies, the data must have a consistent and detailed taxonomy, and be processed and analysed using a consistent methodology^16,17^ Advances in quantitative palaeoecology over recent decades have led to the development of methods to estimate ecological properties of pollen-assemblages^16^, which can then be carefully analysed to draw inferences about past vegetation dynamics^11,13,18–20^.

There have been marked worldwide changes in natural ecosystems during the Holocene in response to natural as well as anthropogenic environmental changes^21–24^. This may have led to significant changes in the phylogenetic relatedness among taxa in the plant assemblages. The incorporation of the time dimension into the spatial analyses of phylogenetic diversity would provide an understanding of the spatio-temporal dynamics of the Holocene-scale phylogenetic assembly of angiosperms along a latitudinal gradient, under changing environmental conditions. So far, studies document inconsistent spatial patterns of phylogenetic relatedness in assemblages per geographical region, groups of organisms, and clades. However, for angiosperms, most empirical studies show an increase in phylogenetic relatedness (= decrease in phylogenetic dispersion (PD)) of assemblages with decreasing temperature and precipitation along increasing elevation or latitude^25,26^. This pattern is referred to as the expectation of the “tropical niche conservatism” hypothesis^27,28^, and suggests that phylogenetic niche conservatism (tendency of taxa or lineages to retain ancestral ecological features and geographic distributions^29^) is a primary mechanism underlying taxon assembly along climatic-latitudinal or elevational gradients. Most of these studies use modern assemblage data only, which limits our understanding of how taxon dispersal, colonisations, and range dynamics in response to spatio-temporal environmental variation, may have influenced the phylogenetic assembly of co-occurring taxa across time and space. Palaeoecological studies of phylogenetic relationships among co-occurring taxa can provide this perspective as they add the time dimension to the analyses of the patterns and their potential drivers and mechanisms^3,9^.

We use Holocene (past ca. 12,000 years) fossil pollen data covering the latitudinal gradient in central Asia to analyse latitudinal and temporal variation in phylogenetic relatedness of angiosperm families in pollen assemblages, which we quantify in terms of average phylogenetic dispersion (PD) in the assemblages. We use two robust and widely recognised metrics of PD that quantify PD at different depths (phylogeny tip-weighted versus phylogeny base-weighted (see methods ‘Phylogenetic diversity metrics’ section)) of evolutionary history. Specifically, we use standardised measures (called ‘standardised effect size’ or ‘ses’) of the mean pairwise phylogenetic distance (MPD) and the mean nearest taxon distance (MNTD) to quantify the PD of taxa on a phylogenetic tree (assemblage) relative to a phylogeny of an appropriate species pool^6^. Higher (positive) values of PD (sesMPD or sesMNTD) in an assemblage along the gradient indicate that taxa in that assemblage are phylogenetically overdispersed (more distantly related) than those expected under a null model of random assemblage composition, whereas low values indicate phylogenetic clustering (closer relatedness) of taxa in the assemblage than those expected under a null model of random assemblage composition^6,30^. In addition, we explore correlations between the spatio-temporal variation in the estimates of these metrics and climatic variables.

Here, we specifically ask:

1. what is the latitudinal pattern in phylogenetic dispersion of angiosperms in central Asia during the Holocene?
2. does the latitudinal phylogenetic dispersion pattern of angiosperms vary temporally during the Holocene?
3. are the spatio-temporal variations in phylogenetic dispersion of angiosperms concordant with millennial scale climatic changes during the Holocene?

**Figure 1:**
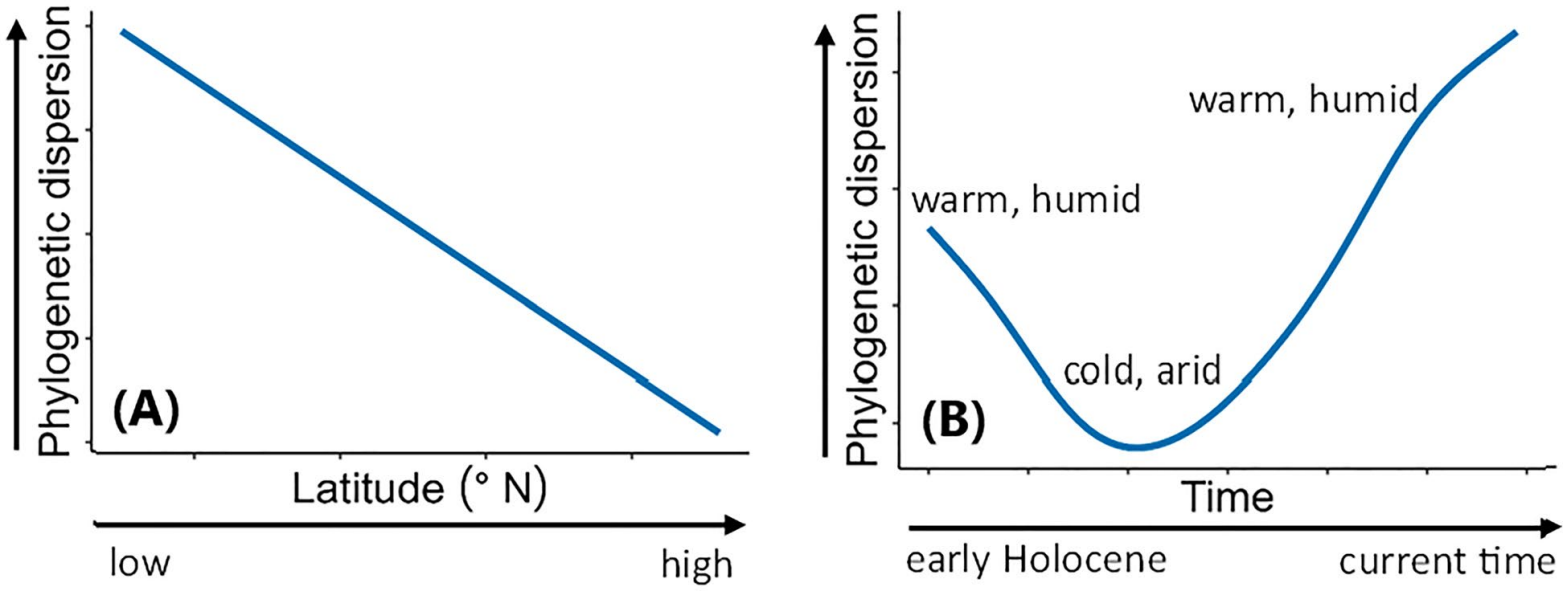
Diagrammatic representation of expected spatio-temporal variation in phylogenetic dispersion (PD) of angiosperms in central Asia during the Holocene. (**A**) Expected spatial (latitudinal) pattern in PD and (**B**) expected temporal pattern in PD.

Spatially, we expect a continuous decrease in PD in angiosperm assemblages with increasing latitude, where PD will decrease progressively with a decrease in temperature, precipitation, and length of growing season (increasing environmental stress) along the latitudinal gradient (Fig. 1A). If the mechanism of phylogenetic niche conservatism holds true, a gradient of increasing environmental stress, i.e., a decrease in temperature, precipitation, and length of growing season with increasing latitude, would filter taxa in the assemblages by limiting dispersal, shift, and colonisation of plant species towards higher latitudes, while favouring only the taxa which have evolutionarily developed cold and drought adaptive characteristics. Co-occurrence of taxa with similar eco-evolutionary features most likely results in phylogenetic clustering, and thus PD in the assemblages is decreased.

Temporally, we assume significant millennial scale variations in latitudinal pattern in PD in response to major environmental variations during the Holocene. Warmer and wetter phases are assumed to have facilitated plant dispersal, shifts, and colonisations, thereby allowing co-occurrence of taxa of different ecological niche leading to an increase in PD of assemblages (Fig. 1B). However, cold, and dry phases would have induced filtering of species in assemblages, allowing only the co-occurrence of cold- and aridity-adapted taxa, and limiting dispersal, range expansion, and colonisation of species into new areas. This would result in a decrease in PD in assemblages (Fig. 1B). These climate driven temporal changes in PD are not expected to be uniform throughout the latitudinal gradient because the timing, frequency, and intensity of the Holocene environmental changes have not been consistent throughout the latitudinal gradient.

## Results

### Spatio-temporal variation in phylogenetic dispersion

**Figure 2:**
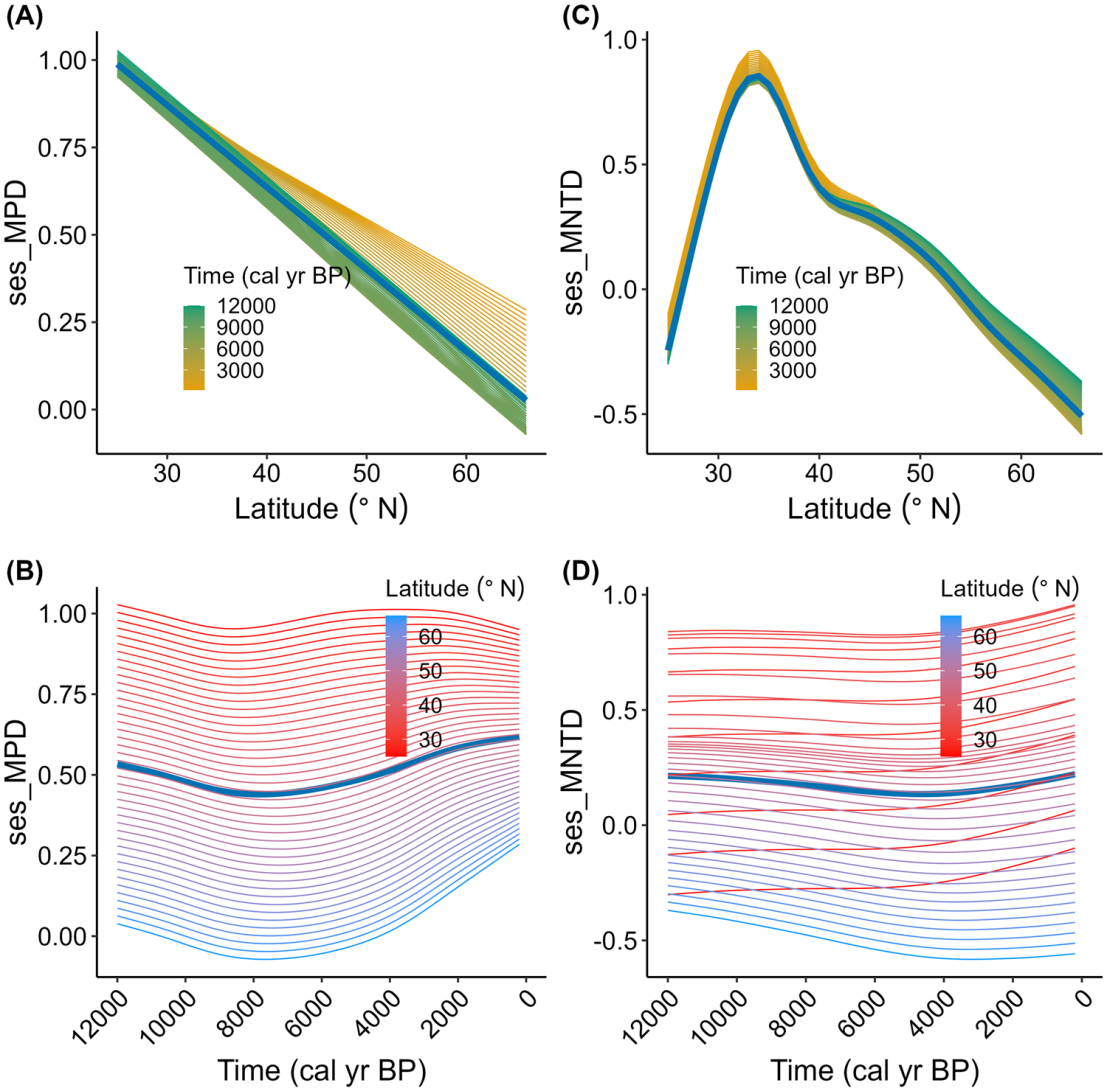
Spatial and temporal patterns of phylogenetic dispersion PD during the Holocene. Standardised effect size of mean pairwise phylogenetic distance (ses_MPD): (**A**) overall latitudinal pattern during the Holocene, and (**B**) spatio-temporal variation; and standardised effect size of mean nearest taxon distance (ses_MNTD): (**C**) overall latitudinal pattern during the Holocene, and (**D**) spatio-temporal variation of angiosperm families in central Asia. Each curve for both metrics represents variation in the metrics for every (**A**, **C**) 200-year time interval and (**B**, **D**) 1° latitude. A statistical summary of the models is presented in Tables 1, 2. ‘cal yr BP’ = calibrated years before present. Detailed figures with the individual data points are presented in Fig. S1.

There is considerable variation in the estimates of PD across space as well as time (Figs. 2, S1). Among the 99 datasets distributed between 25.77° N and 64.83° N latitudes, sesMPD and sesMNTD range from − 1.64 to 3.06 (mean sesMPD = 0.56) and − 2.03 to 3.55 (mean sesMNTD = 0.27), respectively, with sample-age ranging from 1 to 12,000 cal yr BP. For both metrics, variation in the estimates increases along the latitudinal gradient (Fig. 2A,C). However, variation in sesMPD decreases and that in sesMNTD increases temporally from ca. 12,000 years to present (Fig. 2B,D). Overall, both metrics reveal more overdispersion by magnitude as well as by number of assemblages exhibiting overdispersion (PD estimates for the majority of assemblages are greater than zero) than the phylogenetic clustering (PD estimates are negative) (Fig. S1).

### Latitudinal pattern in phylogenetic dispersion

The overall latitudinal pattern of PD in angiosperm families contrasts between sesMPD and sesMNTD at lower latitudes (below ca. 35° N) (Fig. 2A,C). While sesMPD decreases linearly along the latitudinal gradient (Fig. 2A), sesMNTD increases from ca. 25° N to 35° N, and then decreases linearly with increasing latitude (Fig. 2C). Despite this, estimates of both metrics, in general, are higher for assemblages at lower latitudes than those from high latitudes throughout the Holocene (Fig. 2). These latitudinal patterns are statistically significant (adjusted R^2^ = 0.823, 0.811; deviance explained = 0.834, 0.822; p_s(latitude)_ < 0.001, < 0.001, respectively for sesMPD and sesMNTD, Tables 1, 2).

**Table 1:** Statistical summary of hierarchical generalised additive models (hGAM) for the Holocene-wide latitudinal pattern in standardised effect size of mean pairwise phylogenetic distance (sesMPD).

sesMPD						
Component	Term	Estimate	Std error	t-value	*p* value	Sig. codes
Parametric coefficients	(Intercept)	0.60	0.07	7.97	< 0.001	***

Component	Term	edf	Ref. df	F-value	*p* value	
Smooth terms	s(lat)	1.00	1.00	9.00	0.002	**
	s(age)	3.62	4.36	5.97	< 0.001	***
	s(dataset_id)	94.69	97.00	204.23	< 0.001	***
	ti(lat,age)	2.79	3.37	2.46	0.04	*

Std error = standard error, Sig. codes = significance codes, ti = tensor product of the variables within parentheses, edf = estimated degrees of freedom, Ref. df = reference degrees of freedom used in computing test statistic and *p*-values.

Sig. codes: ‘***’ < 0.001 < ‘**’ < 0.01 < ‘*’ < 0.05 < ‘.' < 0.1 < ‘ ' 1, adjusted R-squared: 0.823, deviance explained 0.834, N: 6557.

**Figure 3:**
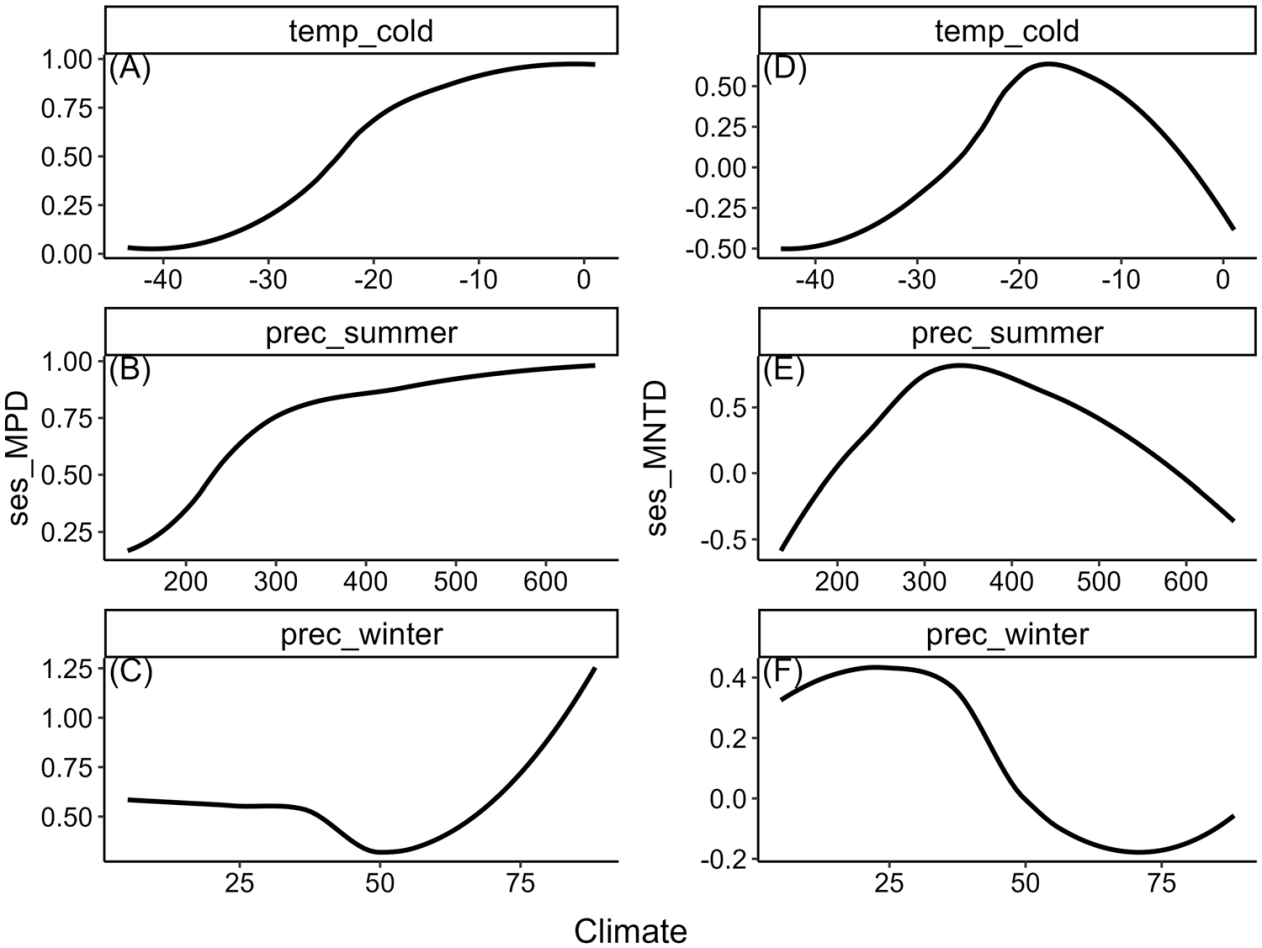
Relationship between latitudinal patterns in phylogenetic dispersion—PD (standardised effect size of mean pairwise phylogenetic distance (sesMPD), standardised effect size of mean nearest taxon distance (sesMNTD)) and climatic variables during the Holocene. (**A**–**C**) sesMPD and (**A**) ‘temp_cold’, (**B**) ‘prec_summer’, (**C**) ‘prec_winter’, (**D**–**F**) sesMNTD and (**D**) temp_cold, (**E**) ‘prec_summer’, (**F**) prec_winter. Here, predictions of the spatio-temporal models (hierarchical generalised additive models hGAMs) for PD are treated as response variables, and predicted values of the spatio-temporal hGAMs for each of the climatic variables are treated as predictor variables (Supplementary text S1B). The black curve is fitted as a ‘loess’ smoother. Climate variables are (**A**) temp_cold = minimum temperature of the coldest month (°C), (**B**) prec_summer = summer precipitation (kilogram meter^−2^ quarter^−1^), (**C**) prec_winter = winter precipitation (kilogram meter^−2^ quarter^−1^)). Statistical significance of these relationships was tested by a Procrustes randomization test (Table 3).

Temporally, the overall patterns of PD partly differ for sesMPD and sesMNTD at higher latitudes (blue curves in Fig.  2B,D), where both decrease gradually from the LGM-Holocene transition to the mid-Holocene, and then sesMPD increases whereas sesMNTD decreases from the mid-Holocene to present. At lower latitudes south of ca. 45° N (red curves in Fig. 2B,D), sesMPD decreases gradually from the LGM-Holocene transition to the mid-Holocene, whereas no temporal changes are evident for sesMNTD during this period. However, from the mid-Holocene to present, there is a gradual increase in the estimates of both metrics (Fig. 2B,D). Notably, there is a large variation in the estimates of sesMNTD at lower latitudes (Fig. 2D). There is a statistically significant concordance between spatio-temporal variation in each of the PD metrics and the climatic factors except winter precipitation along the latitudinal gradient (Fig. 3, Table 3).

**Table 2:** Statistical summary of hierarchical generalised additive models (hGAM) for the Holocene-wide latitudinal pattern in standardised effect size of mean nearest taxon distance (sesMNTD) of angiosperm families in Asia.

sesMNTD						
Component	Term	Estimate	Std Error	t-value	*p* value	Sig. codes
Parametric coefficients	(Intercept)	0.35	0.06	5.13	< 0.001	***

Component	Term	edf	Ref. df	F-value	*p* value	
Smooth terms	s(lat)	4.83	4.86	7.54	< 0.001	***
	s(age)	2.94	3.52	2.75	0.03	*
	s(dataset_id)	89.98	97.00	81.14	< 0.001	***
	ti(lat,age)	1.00	1.00	4.59	0.03	*

Std error = standard error, Sig. codes = significance codes, ti = tensor product of the variables within parentheses, edf = estimated degrees of freedom, Ref. df = reference degrees of freedom used in computing test statistic and *p*-values.

Sig. codes: ‘***’ < 0.001 < ‘**’ < 0.01 < ‘*’ < 0.05< ‘.' < 0.1 < ‘ ' 1, adjusted R-squared: 0.811, deviance explained 0.822, N: 6557.

### Temporal variation in latitudinal pattern of phylogenetic dispersion

**Figure 4:**
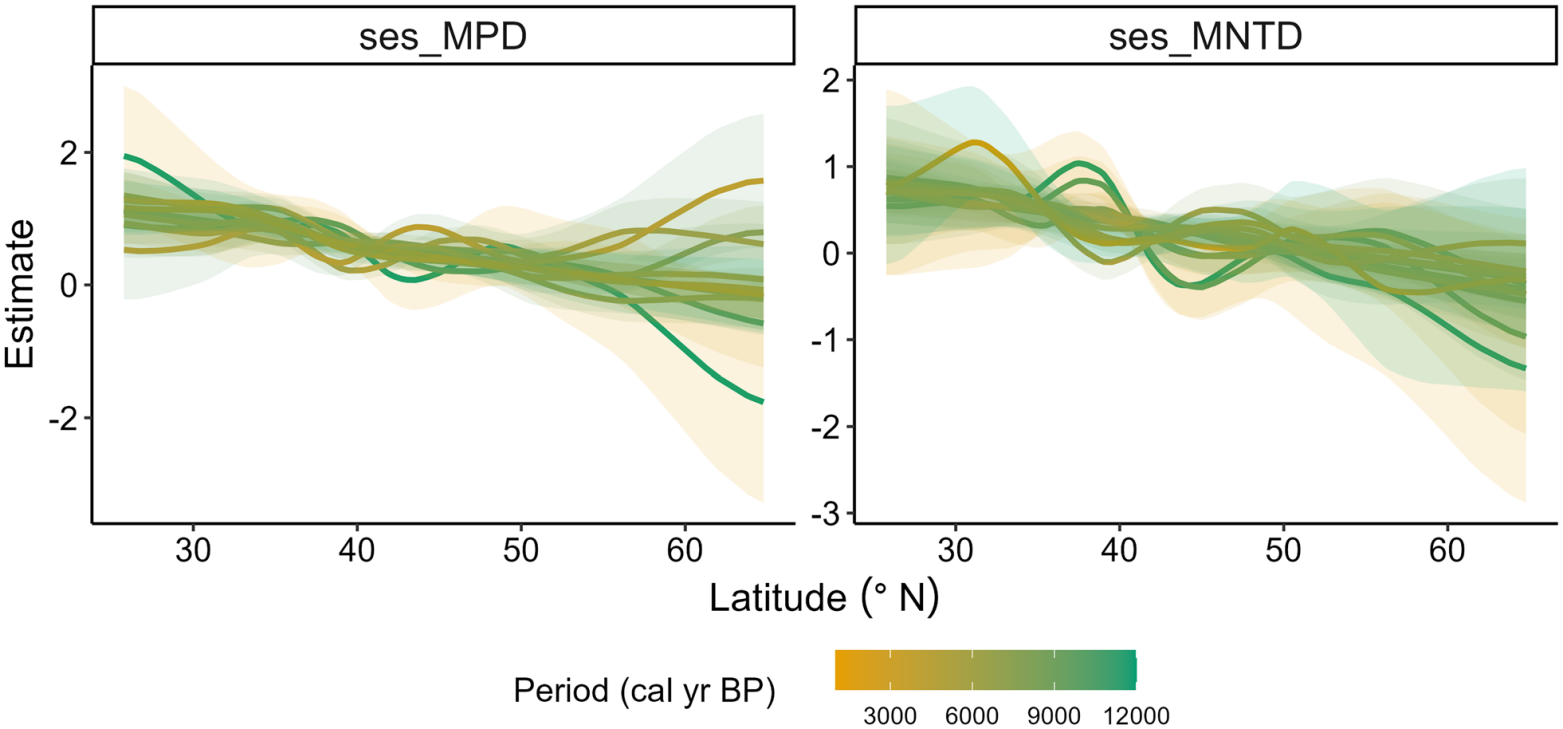
Temporal (millennial) variation in the latitudinal pattern of phylogenetic dispersion—PD (standardised effect size of mean pairwise phylogenetic distance (sesMPD), standardised effect size of mean nearest taxon distance (sesMNTD)). Curves of the latitudinal pattern are predicted by a hierarchical generalised additive model, where PD is treated as a response variable: latitude (lat), sample-age (age), and the tensor product of latitude and age are fixed-effect variables; and location (factorial) and period (factorial) are treated as random-effect variables. Gradient of colour of the curves represents the pattern for every 1000-year period. ‘cal yr BP’ = calibrated years before present. This model is different from that for the Holocene latitudinal pattern (Fig. 2A,C) in that there is no global smoother of latitude in this model, and therefore, the latitudinal pattern of PD for each period has its own wiggliness (smoother) and penalty, thereby allowing comparison of the smooths (slopes) of the models for each period between each other. A statistical summary of the models is presented in Table S1.

**Figure 5:**
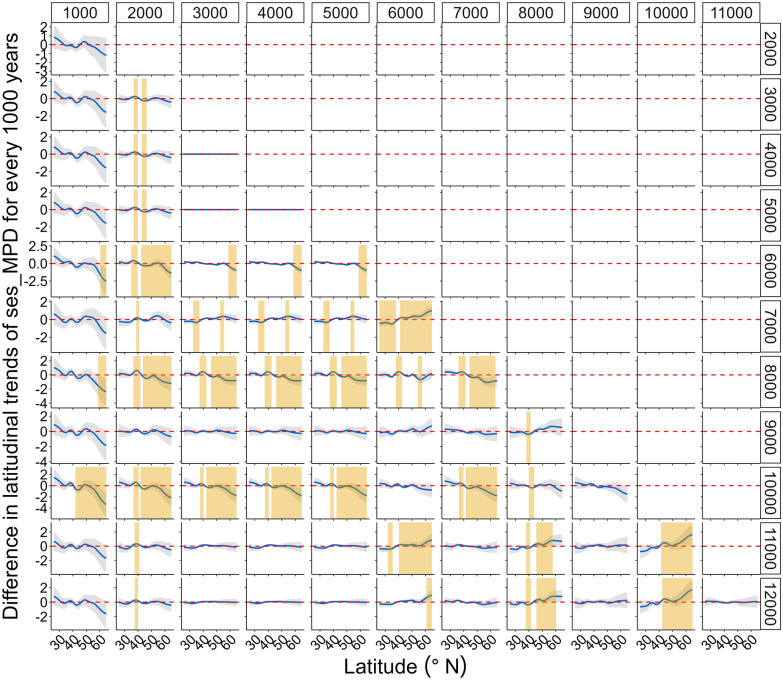
Spatio-temporal variation in the standardised effect size of mean pairwise phylogenetic distance (sesMPD). Here, smooths of the latitudinal pattern of sesMPD (hierarchical generalised additive models) for each 1000-year period are compared to each other. Complete deviation of the 95% confidence interval (CI) (i.e., CI (grey) does not include the zero-threshold (dashed red line)) and departs from the threshold line by at least ± 0.05 through at least 2° continuous variation in latitude along the latitudinal gradient. Periods shaded with yellow colour show significant temporal variation in the pattern at that latitude.

**Figure 6:**
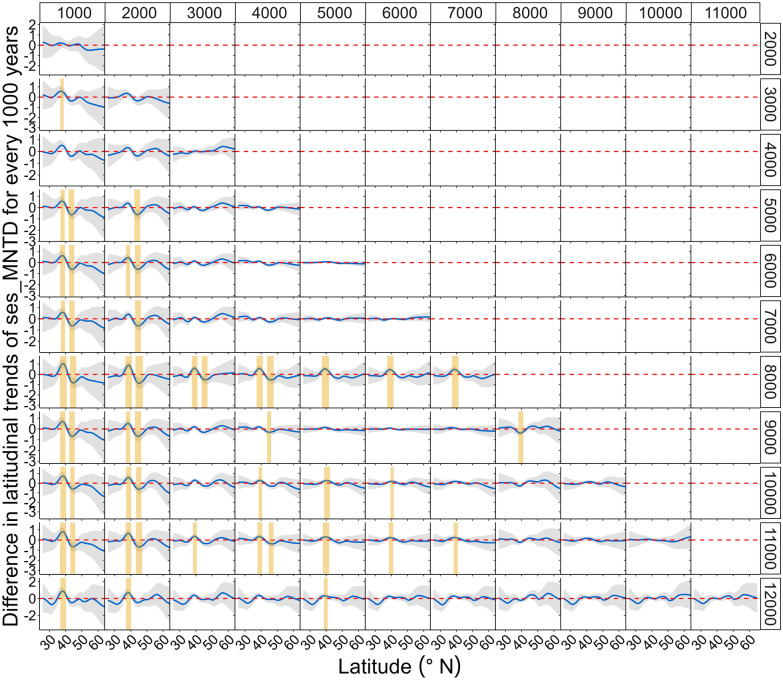
Spatio-temporal variation in the standardised effect size of mean nearest taxon distance (sesMNTD). Here, smooths of the latitudinal pattern of sesMNTD (hierarchical generalised additive models) for each 1000-year period are compared to each other. Complete deviation of the 95% confidence interval (CI) (i.e., CI (grey) does not include the zero-threshold (dashed red line)) and departs from the threshold line by at least ± 0.05 through at least 2° continuous variation in latitude along the latitudinal gradient. Periods shaded with yellow colour show significant temporal variation in the pattern at that latitude.

Coarsely, there is no distinct variation in the latitudinal patterns of the PD metrics for different time periods, as the curves and 95% confidence intervals for each time period are not clearly differentiated (Fig. 4). However, closer examination of the difference in the smooths (slopes) of the models of latitudinal patterns across periods reveals that the temporal patterns of both metrics change significantly at particular latitudes across particular time-periods (Figs. 5, 6). Spatially, a significant temporal variation in the latitudinal pattern of sesMPD is observed at mid- (ca. 35° N) to high-latitudes (ca. 65° N) (Fig. 5), whereas that of sesMNTD is evident at mid-latitudes (ca. 35° N–45° N) (Fig. 6). Temporally, significant variation in the latitudinal pattern of sesMPD and sesMNTD is mostly observed in the 2000–1000 year, 8000–6000 year, and 11,000–10,000 year time periods (Figs. 5, 6; Table S1). A Procrustes randomisation test between spatio-temporal variation in PD and in each of the climatic variables reveals statistically significant concordance in the spatio-temporal variation of PD and climate (minimum temperature of coldest month ‘temp_cold’ and precipitation of the warmest quarter ‘prec_summer’). However, concordance between PD metrics and precipitation of the coldest quarter (‘prec_winter’) is not statistically significant (Table 3).

**Table 3:** Procrustes randomisation test (PRT) between spatio-temporal variation in the measures of phylogenetic dispersion—PD (standardised effect size of mean pairwise phylogenetic distance (sesMPD), standardised effect size of mean nearest taxon distance (sesMNTD)) and spatio-temporal variation in each of the climatic variables at the same spatial and temporal resolution as for PD.

Matrix_1 (PCA)	Matrix_2 (PCA)	Proc sum sq	Corr proc rot	Significance	N_rand	Sig. codes
sesMPD	temp_cold	0.85	0.38	< 0.001	999	***
	prec_summer	0.62	0.61	< 0.001	999	***
	prec_winter	0.99	0.04	0.92	999	
sesMNTD	temp_cold	0.71	0.53	< 0.001	999	***
	prec_summer	0.55	0.67	< 0.001	999	***
	prec_winter	0.99	0.003	0.99	999	

Principal component analysis (PCA) was performed with standardized matrices of spatio-temporal variation of each variable, and the resulting matrices were then used in PRT. ‘temp_cold’ = minimum temperature of the coldest month, ‘prec_summer’ = summer precipitation, ‘prec_winter’ = winter precipitation. Proc sum sq = Procrustes sum of squares (m^2^), Corr Proc rot =  correlation in a symmetric Procrustes rotation, N_rand =  number of randomizations, Sig. codes = significance codes.

Significance codes: ‘***’ < 0.001 < ‘**’ < 0.01 < ‘*’ < 0.05 < ‘ .' < 0.1 < ‘ ' 1.

## Discussion

In this study, 6557 individual assemblages from 99 fossil pollen records, cover a temporal span of 12,000 years and are distributed along a latitudinal range of ca. 40°. We analysed the spatial (latitudinal) and temporal (past 12,000 years) variation in PD of angiosperm families using these assemblages. Our results show that PD varied unevenly along the latitudinal gradient and across time on a millennial scale during the Holocene (Figs. 2, 5, 6). The pattern of this spatio-temporal variation is slightly different for overall PD resulting from evolutionarily deep as well as recent levels of the phylogenetic tree (expressed as sesMPD) compared to evolutionarily recent PD quantified from the phylogeny tips (expressed as sesMNTD) (Figs. 2, 5, 6). Despite this, overall spatio-temporal variation in PD during the Holocene (Fig. 2) is statistically significantly concordant with the Holocene climate dynamics (especially summer precipitation and winter temperature) in central Asia (Fig. 3, Table 3). This is because both the PD metrics exhibit similar latitudinal patterns along most (ca. 35°N–66°N) of the gradient (Fig. 2A,C), where the temporal variations in the metrics are also most pronounced and are broadly similar (Fig. 5, 6). Specifically, the latitudinal pattern in PD varies significantly only from mid- to high-latitudes at certain time periods during the early-, mid-, and late-Holocene (Figs. 5, 6). This implies that the relative influence of the drivers and mechanisms of phylogenetic assembly of angiosperms varied with latitude and time during the Holocene.

The overall Holocene pattern of sesMPD shows a linear decrease in the PD of angiosperm families with increasing latitude (Fig. 2A,B). This pattern is expected considering the global gradient of decreasing temperature, precipitation, length of growing season, increasing temperature seasonality, and strength of winter frost from the subtropics to the pole as documented by several previous studies^31–33^. At the lower latitudes, high environmental and habitat heterogeneity favour assemblages that contain many taxa from phylogenetically distant lineages, thereby increasing PD. However, the above-mentioned environmental factors with increasing latitude most likely hampered the successful dispersal and colonisation of species towards higher latitudes and caused filtering of taxa, allowing co-occurrence of only stress (cold and drought)-tolerant taxa. Central Asia north of ca. 35° N has been influenced mostly by the westerly circulation, which remained generally cold and dry throughout the Holocene^34,35^. Hence, assemblages in the region mostly contain desert, semi-desert, and steppe taxa^36,37^, which have developed cold- and drought-tolerant traits through evolutionary time. Because evolutionarily related taxa tend to have similar ecological tolerances due to the conservative nature of their phylogenetic niche, assemblages in such a stressful environment usually contain phylogenetically closely related taxa, which decreases PD in the assemblages. These drivers and mechanisms also explain the latitudinal gradient in evolutionarily more recent PD in assemblages (sesMNTD) towards higher latitudes (above ca. 35°) (Fig. 2C,D).

However, an increase in sesMNTD from ca. 25° to 35° N is rather unexpected, if we consider the latitudinal variation in major climatic factors along this section of the latitudinal gradient (Fig. S2). This belt mostly falls within the ‘Temperate’ climate zone with a monsoonal climate system (Fig. S3, see methods ‘Holocene climate and vegetation’), where environmental conditions have remained relatively mild throughout the Holocene^35,38^. The strongest distributional and compositional changes in response to spatio-temporal climatic fluctuations during the Holocene are expected to have happened in the transitional zone between the ‘Temperate’ and northern/western ‘Cold-seasonally dry’ zones (Fig. S3, see methods ‘Holocene climate and vegetation’, Bhatta et al.^39^), where sesMNTD also has a peak. Temperate vegetation expanded towards higher latitudes during the warm and humid periods and retreated from these during the cold and dry periods in the Holocene. This zone, with a relatively milder climate, may have acted as a dispersal corridor during warm and humid periods and as a refugium during cold and dry periods. These spatio-temporal vegetation changes in the transitional zone mostly likely facilitated the dispersal and colonisation of some distantly related taxa in this part of the latitudinal gradient. Lower latitudes in this region have also been under significant human influence, for example land-use changes due to agriculture, livestock farming, deforestation, etc., during the Holocene^39–43^. Therefore, we assume that land-use change, in concert with temporal climatic variations, might also have played a role in governing the observed gradient of sesMNTD. However, in the absence of empirical data, we are unable to test the magnitude and direction of such anthropogenic influence on the observed gradient in sesMNTD.

An influence of the Holocene climate dynamics in central Asia and Siberia is also reflected in the temporal patterns of PD along the latitudinal gradient (Fig. 2B,D). Warm and humid climatic conditions in the region during the onset of the Holocene (see methods ‘Holocene climate and vegetation’, Bhatta et al.^39^) most likely caused deglaciations and favoured the dispersal, colonisation, and range expansion of angiosperm taxa throughout the region thereby resulting in phylogenetic diversification of the assemblages. However, the progressively developing cooler and drier conditions from the early- to mid-Holocene most likely caused filtration of taxa, allowing the assembly of only those taxa that were evolutionarily adapted to these environmentally stressful conditions. A marked increase in overall PD (sesMPD) after the mid-Holocene, especially at high latitudes, is concordant with the gradually developing warmer and wetter regional climatic conditions at these latitudes. Increased deglaciations at the higher latitudes facilitated successful dispersal, colonization, and range expansion, especially of the evolutionarily older taxa which were already present in the refugia along mid- to high-latitudes (temperate regions). The absence of a temporal trend in the evolutionarily recent PD (sesMNTD) at high latitudes during the mid- to late-Holocene is probably because most taxa in the assemblages had dispersed relatively recently from the lower latitudes and there has not been sufficient time for further dispersal and colonization of new taxa from lower latitudes.

Compared to the high latitudes, the influence of temporal climatic changes at lower latitudes is not that pronounced on the PD of assemblages because the climatic conditions in these regions were milder and probably did not significantly constrain the phylogenetic assembly of taxa throughout the Holocene. Moreover, anthropogenic activities that started during the mid-Holocene in these regions (see above), most likely caused habitat diversification, thereby facilitating the assembly of taxa with diverse ecological niches in the assemblages. This interplay of climate and anthropogenic influence at lower latitudes resulted in a gradual temporal increase in PD at these latitudes, especially after the mid-Holocene. This trend is evident in both metrics, but especially in the metric which quantifies evolutionarily recent PD towards the tips of the regional phylogeny, i.e., sesMNTD (Fig. 2D).

Temporal variation in the phylogenetic assembly of angiosperms is not consistent through time (12,000–0 cal yr BP) and space (ca. 25° N–66° N). Therefore, there is no clear signal of variation in the latitudinal pattern of PD for different time periods (Fig. 4). However, significant millennial-scale changes in both metrics are observed at mid- to high-latitudes (ca. 35° N–60° N) during the Last Glacial Maximum (LGM)-Holocene transition (ca. 11,000–10,000 cal yr BP), mid-Holocene (ca. 8000–6000 cal yr BP), and late-Holocene (ca. 2000–1000 cal yr BP) (Figs. 5, 6, Table S1). This spatio-temporal variation in PD is broadly concordant with the spatio-temporal patterns of regional climate (especially precipitation regime) and vegetation in central Asia during the Holocene (Fig. S2, see also methods "Holocene climate and vegetation",^39^ and references therein). Low PD in the assemblages under generally low temperature and moisture conditions (Figs. 2; S2 ‘wint_prec’, ‘sum_prec’, ‘temp_cold’) in this region might have resulted in marked spatio-temporal changes in the taxon co-occurrence patterns with further changes in climatic episodes during the Holocene. Notably, the pattern in sesMNTD, which quantifies only the evolutionarily recent PD in the assemblages, reveals significant temporal changes mainly in the mid-latitude region (ca 35–40° N) (Figs. 2C,D, 6), which is the transition zone between the ‘Temperate’ and ‘Arid’ climatic zones (Fig. S3). This possibly reflects a combined effect of climate and anthropogenic land-use dynamics in the region over recent millennia, as indicated by several previous studies^39^.

These spatio-temporal patterns indicate that the phylogenetic assembly of angiosperms along the latitudinal gradient during the Holocene varied temporally at a millennial scale, mostly driven by climatic changes (especially summer precipitation and winter temperature) in central Asia. The cold and dry climatic conditions through space and/or time during the Holocene limited the assemblage composition mostly to stress-tolerant (low temperature and moisture) taxa along the latitudinal gradient and caused filtration or displacement of other taxa due to ecological niche conservatism of the taxa. The physiological tolerances of a taxon to climatic extremes determine the elevational and latitudinal limits of that taxon^44^ and hence, limit the taxa as well as phylogenetic diversity. Previous studies have also documented that the temperature- and precipitation-related climatic extremes, as used in this study, are important in driving the phylogenetic diversity of different groups of organisms^45–47^. Thus, they support the importance of mechanisms such as phylogenetic niche conservatism, and environmental filtering for the phylogenetic assembly of angiosperms along the latitudinal gradient in central Asia.

To conclude, PD among co-occurring angiosperm taxa generally decreased during the Holocene with increasing environmental stress along the latitudinal gradient in Asia. This implies that environmental filtering due to phylogenetic niche conservatism is the main driving eco-evolutionary mechanism underlying the phylogenetic assembly of angiosperms along the latitudinal gradient. An increase in evolutionarily recent PD (sesMNTD) from low- to mid-latitude is most likely a phylogenetic response of assemblages to spatio-temporal dynamics of regional climate. Temporally, PD among co-occurring taxa has changed significantly at a millennial scale during the Holocene, especially in the environmentally stressful areas along mid- to high-latitudes. Thus, the relative influence of the mechanisms governing the phylogenetic assembly of angiosperms along the latitudinal gradient varied with space (latitude) and time during the Holocene. This latitude-dependent response of PD is largely concordant with Holocene climate dynamics. This indicates that the major environmental changes during the Holocene were strong enough to drive phylogenetic changes in the angiosperm assemblages, especially in those with low PD. This sensitivity predicts that ongoing environmental changes of the same or higher magnitude and frequency may bring further profound phylogenetic changes in the plant assemblages in the environmentally stressful high-latitude areas.

## Materials and methods

### Study area

We compiled 99 fossil-pollen records from central Asia between 25° N–66° N latitude and 75° E–125° E longitude (Fig. S3). Records outside this area are excluded to avoid potential variation in pollen composition due to major mountain ranges such as the Himalaya in the south and the Urals in the west, and to the oceanic, polar, and inter-continental climatic systems. Spatially, records are distributed from the “Temperate” (south Asia) to subpolar “Cold without dry season” climatic zone in the north^cf.^^48^, representing variation in climate and vegetation along a latitudinal range of ca. 40°. Based on the climatic zonation of Beck et al*.*^48^, part of the high Tibetan Plateau is classified in the ‘Polar’ climate-zone based on its topography, temperature, and precipitation regimes (Fig. S3). Temporally, the age of the youngest sample (upper age-limit) ranges from 0 to 1600 cal yr BP (calibrated years before present, where 0 yr BP = 1950 CE) and that of the oldest sample (lower age-limit) varies from 5900 to 12,600 cal yr BP. The distribution of samples and records is fairly homogeneous across time and space (Fig. S4). Of the 99 records, 87 cover at least 8000 years within the Holocene. Thus, these data provide a good representation of the temporal extent of the Holocene. Compilation and processing of these records are described in Bhatta et al*.*^39^ and Flantua et al*.*^49^.

### Holocene climate and vegetation

Based on temperature and precipitation regimes, the current climate in our study area can be summarised into five Köppen-Geiger climate zones or ‘broad biomes’^cf.^^48^ (Fig. S3). These are Arid (north-western parts of the Indian Subcontinent, parts of Mongolia including the Gobi desert, and north-west China); Cold-seasonally dry (parts of north-east China and Mongolia and south-east Siberia); Cold without dry season (regions between 50° N and 66° N latitudes, i.e. central Siberia); Temperate (northern and north-eastern parts of southern Asia); and Polar (northern parts of Siberia and high elevation areas in central and southern Asia)^48^ (Fig. S3). South-west Asia and central Asia experience an arid or semi-arid climate influenced by the Mediterranean climate system, whereas the monsoon climate system dominates across southern and eastern regions (Fig. S3 ‘Temperate’)^35^. This results in a marked precipitation gradient from southern and south-east Asia to the desert of central Asia (Fig. S3 ‘Arid’) where precipitation is influenced by the westerlies^34^. The eastern parts are influenced by the Indian summer monsoon, the East Asian summer monsoon, and the westerly circulation^34^. This spatial variation in precipitation has resulted in a prominent precipitation gradient from monsoon-dominated south-eastern Asia (Fig. S3, “Temperate”) to the desert areas of central Asia that are influenced by the westerlies (Fig. S3, “Arid”)^34^. The Siberian region is influenced by the polar climate system and the ‘Siberian High’, with low temperature and precipitation^50^. Overall, Asia at present is mild to hot and humid in the south, very cold and dry in the far north and north-east, and temperate (moderate temperature and with or without aridity) at mid-latitudes (Fig. S2A,C).

The Holocene climate in Asia shows considerable variation across space and time (Fig. S2). In general, early- to mid-Holocene climate in eastern and central Asia became warm and humid as temperature and summer monsoon activity increased, and then became cooler and drier from the mid- to late-Holocene as precipitation decreased^38,51^. Temperature increased steeply over recent centuries^38^. In arid central Asia, the early-Holocene was cold and dry, but warmer conditions progressively developed during the mid- to late-Holocene^35^. In Siberia, the early- to mid-Holocene was warmer and wetter than today becoming drier and cooler by the late-Holocene, especially in western Siberia. Most present-day Siberian ecosystems started to develop after ca. 3000 cal yr BP. There has been a recent rapid rise in temperature and increase in winter precipitation north of 55° N^52^.

The vast variation in physiography and climate of Asia today is reflected by its diverse vegetational patterns. Current vegetation in the eastern parts follows the south–east–north–west precipitation gradient. It varies from a moist coastal-forest zone to steppe/desert, consisting of broad-leaved, deciduous and boreal coniferous forests, high meadow, steppe, semi-desert, and desert species^53,54^. Central Asia contains Irano-Turanean and Central-Asiatic floristic elements, mainly with desert, semi-desert, and steppe vegetation^55^. Arid vegetation in this region mainly occupies low elevations, whereas grassland, shrub, and forest predominate at mid-elevations on mountain slopes, and meadow and tundra-like ecosystems exist at high elevations. In the north, coniferous forests form a transition zone between Siberian taiga forest and central Asian steppe^55^. Siberian vegetation shows latitudinal vegetation zones^56^. The tundra zone occupies the northern part, where the high Arctic tundra consists of herb-lichen-moss associations, whereas central and southern tundra is dominated by low-shrub species. The taiga zone extends between c. 50° N and 70° N latitude, mainly in western and central Siberia. It is dominated by conifers and with scrub and woodlands of *Betula* and *Salix*. Forest-steppe and meadow steppe zones occur in the southern part of western Siberia^52^.

Studies suggest that spatio-temporal changes in the vegetation of Asia were very heterogenous during the Holocene^39^. A general pattern is that there was the dominance of cold- and drought-resistant steppe vegetation at the onset of the Holocene, which was replaced by an increase of forests in parts of central Asia, eastern Asia, and southern Siberia from the early- to mid-Holocene, likely in response to increased temperature and precipitation^54^. Low latitude/altitude regions in eastern Asia were colonized by thermophilous broadleaved forest during the early-Holocene, and the forest expanded westwards and northwards until the mid-Holocene, in response to optimal regional climatic conditions^54^. However, during the mid- to late-Holocene, mixed forests declined regionally and coniferous vegetation with dominant taxa such as *Abies, Picea,* and *Pinus* increased, probably in response to changes in land-use and regional climate, especially the weakening of the summer monsoon^42,57^. On the eastern and north-eastern parts of the Tibetan plateau *Picea-Betula* forest flourished in response to increased humidity conditions during the early-Holocene, and then decreased regionally and was replaced by alpine steppe vegetation as conditions became drier during the mid- to late-Holocene^58^. In high-elevation areas of central Asia, temperate- and alpine-steppe vegetation was dominant during the early-Holocene. This was replaced by taiga or high-alpine meadows during the mid-Holocene due to changes in temperature and moisture conditions, resulting in a significant decline in forests regionally. However, forest in the low-elevation basins and plains increased after the mid-Holocene due to increased humidity^37,59^. The northern and western parts of Tibetan plateau were dominated by temperate desert vegetation throughout the Holocene. In the north-eastern Siberia, the early-Holocene was represented by cold and drought-resistant tundra and steppe plant communities. A progressively developing warmer and humid climate during the early-Holocene favoured the replacement of tundra–steppe vegetation by *Picea*-*Larix* forest-tundra with a shrub understory. In southern Siberia, forest cover increased during the early-Holocene in response to a significant rise in temperature and moisture, whereas in the northern Arctic belt, *Betula*-*Larix* forests dominated during the early- to mid-Holocene. Climate became progressively cooler and drier during the mid-Holocene, and this resulted in the replacement of forest-steppe by cold- and drought-tolerant *Betula* forest, the southward retreat of relatively thermophilous *Abies* forests, lowered treelines in the high mountain areas of the Altai, and the freezing of wet mires. A continuous cold and dry phase in Siberia during the late-Holocene (except the recent past centuries) resulted in a southward retreat of forest to reach its present position^37,52,60^. A detailed account of Holocene climate and vegetation in Asia is presented in Bhatta et al*.* ^39^.

### Data compilation

Of the 193 processed pollen records from Asia and Siberia^cf.^^39^, 99 lie within the pre-defined range of latitude and longitude (Fig. S3). The datasets include pollen of terrestrial trees, shrubs, herbs, palms, succulents, and mangrove taxa. Each dataset has five or more samples, and more than five pollen taxa. We filtered the samples based on the number of pollen grains in each sample of a dataset. If the pollen count was consistently low (more than 50% of samples in a dataset had < 150 pollen grains) in a record, we discarded the samples containing < 25 grains. However, if 50% of the samples in a record had > 150 grains, we filtered-out the samples with < 150 grains. Filtering of the samples based on this criterion was not possible for the data that are only available as pollen-percentages. For each record, we compiled information about latitude, longitude, site name or location, country, sample depth, number of samples, depositional environment, current climate based on the classification of Beck et al*.*^48^, and relevant publications. Altogether, 6557 samples (assemblages) from the 99 processed pollen records are used for analyses. For more detailed information about selection and processing of fossil pollen records, see Flantua et al*.*^49^.

### Age-depth models

For estimating the age of pollen samples in each record, we constructed a chronology for each sequence using the Bchron age-depth modelling approach in R, with 50,000 iterations^61^. Age-depth models for all pollen records are available at https://github.com/HOPE-UIB-BIO/Latitudinal_phylodiversity_publication. We treated the median of the calibrated age (cal yr BP) of each sample as the sample-age (hereafter ‘age’), and its 95% confidence interval as an associated uncertainty in the age estimation. We used this age-uncertainty to derive an ‘age-uncertainty index’ for each sample in each record and used this index as a weight in all the numerical modelling, so that a sample with a smaller age-uncertainty received more weight compared to one with a larger age-uncertainty. We estimated the index as follows:

age-uncertainty index of a sample = Mean difference between the upper and lower 95% confidence intervals of the calibrated age for all samples in all records/Difference between the upper and lower 95% confidence intervals of the calibrated age for the sample.

The spatio-temporal distribution of the age uncertainty of all samples is presented in Fig. S5. Early-Holocene samples (c.12000–7500 cal yr BP) around 45° N and 50° N have higher age uncertainties than other samples.

### Taxonomic harmonisation of fossil pollen

Fossil pollen taxa are pollen morphotypes identified by palynologists based on the morphology of pollen found in the sediment samples. Nomenclature and level of identification may differ among sequences and among analysts^62^. Pollen types are sometimes identifiable to the species or genus level but most often a family can only be identified with certainty. Construction of a species phylogeny based on these morphotypes is therefore not possible. Therefore, we harmonised the pollen taxonomy by merging all pollen morphotypes into their respective families. We followed the latest classification system of Angiosperm Phylogeny Group (APG IV)^63^ to assign plant families to pollen morphotypes because the most updated time-calibrated phylogenies of spermatophytes are based on the nomenclature and classification system of APG IV. Botanical nomenclature of the families was standardized according to the World Flora Online (http://www.worldfloraonline.org/, accessed on 2023-09-28).

We harmonised 1955 ‘raw’ pollen taxa into 153 harmonised angiosperm families (https://github.com/HOPE-UIB-BIO/Latitudinal_phylodiversity_publication). Thus, although this process standardises the fossil pollen datasets and makes the results of analyses comparable and interpretable across multiple datasets, it should be emphasized that any phylogenetic diversity existing among taxa at lower taxonomic levels (genus, species, or subspecies) is averaged to family-level phylogenetic variation.

### Construction of family-level phylogeny

We used the time‐calibrated maximum likelihood angiosperm family‐level phylogeny (Data1_RAxML.tre) by Ramírez-Barahona et al*.*^4^ as a backbone tree which includes 437 families of angiosperms in the world. To the best of our knowledge, this is the most updated phylogeny for angiosperm families. All, except family Viburnaceae, 153 plant families in our data (regional pool) are found in the backbone tree. We renamed Viburnaceae to its synonym Adoxaceae to match all the families in our data with those in the backbone tree, and then directly pruned the tree to construct the regional phylogeny by retaining only families present in the regional pool. We used the ‘ape’ package^64^ in R version 4.2.0^65^ to construct the regional phylogeny.

### Phylogenetic diversity metrics

We used two commonly used metrics of PD, namely mean pairwise phylogenetic distance (MPD) and mean nearest taxon distance (MNTD) (Eqs. 1–3)^6,66^. There are two main advantages of these measures over other metrics of phylogenetic diversity. First, these metrics can be standardized (standardized effect size ‘ses’) to the mean and expectation of the mean pairwise distance given the tree (phylogeny of regional pool) and taxon richness of the sample (Eq. 1), which accounts for differences in taxon richness among assemblages analysed and reveals the average phylogenetic relatedness of a pair of taxa in an assemblage^6,66,67^.

1 $$ {\text{sesMPD}}/{\text{sesMNTD}} = \frac{{({\text{MPD}}/{\text{MNTD}}_{{{\text{observed}}}} - {\text{ MPD}}/{\text{MNTD}}_{{{\text{randomised}}}} )}}{{{\text{sdMPD}}/{\text{MNTD}}_{{{\text{randomised}}}} }} $$

where MPD/MNTD_observed_ is the observed MPD/MNTD within an assemblage, MPD/MNTD_randomised_ is the expected MPD/MNTD of the randomised assemblages (n = 9999 randomisations), and sdMPD/MNTD_randomised_ is the standard deviation of the MPD/MNTD for the randomised assemblages.

Second, MPD is estimated as the standardized measure of mean pairwise phylogenetic distance (sesMPD) (Eq. 2) among all possible pairs of taxa in an assemblage across the tree, rather than weighting basal or tip divergences more heavily^68^. Therefore, it is particularly suited to measure overall PD resulting from a deep level as well as recent clustering or overdispersion.

2 $$ {\text{MPD}} = \frac{{\mathop \sum \nolimits_{{{\text{i}} = 1}}^{{{\text{N}} - 1}} \mathop \sum \nolimits_{{{\text{j}} = {\text{i}} + 1}}^{{\text{N}}} {\text{d}}_{{{\text{ij}}}} {\text{P}}_{{\text{i}}} {\text{P}}_{{\text{j}}} }}{{\mathop \sum \nolimits_{{{\text{i}} = 1}}^{{{\text{N}} - 1}} \mathop \sum \nolimits_{{{\text{j}} = {\text{i}} + 1}}^{{\text{N}}} {\text{P}}_{{\text{i}}} {\text{P}}_{{\text{j}}} }} $$

where d_i,j_ is the phylogenetic distance between taxon *i* and *j*, and P_i_ and P_j_ are the relative abundance of taxon *i* and *j*.

On the other hand, MNTD represents a standardized measure of the mean phylogenetic distance of a taxon to its nearest taxon within a sample (sesMNTD) (Eq. 3), and is therefore best suited to quantify PD towards the tips (recent) of the regional phylogeny pool, independent of deep-level clustering^68^.

3 $$ {\text{MNTD}} = \user2{ }\frac{{\mathop \sum \nolimits_{{{\text{i}} = 1}}^{{\text{N}}} {\text{min}}\left( {\mathop \sum \nolimits_{{{\text{j}} = 1}}^{{{\text{N}}\left( {{\text{j}} \ne {\text{i}}} \right)}} {\text{d}}_{{{\text{ij}}}} {\text{P}}_{{\text{i}}} {\text{P}}_{{\text{j}}} } \right)}}{{\text{N}}} $$

Thus, despite being often strongly and positively correlated, both metrics capture a different part of the unique phylogenetic information in an assemblage. We estimated sesMPD and sesMNTD for each sample in the datasets using the ‘picante’ package in R^67^, which provides computationally optimal and robust estimates of both metrics and allows comparisons of PD among assemblages that vary in taxon richness^67^.

In our data, each sample from a fossil pollen record represents an assemblage. We randomised each assemblage by drawing a taxon (family) from the pool of phylogeny with the family occurring in at least one assemblage (sample pool) with equal probability, while maintaining per-sample family richness and weighting the families by their total abundances in the record. The regional phylogeny pool used in these randomisations includes all the families occurring in the 193 datasets from Asia and Siberia. Increase in PD (sesMPD and sesMNTD) in an assemblage along the latitudinal gradient indicates phylogenetic overdispersion, i.e., co-occurring taxa are distantly related than those expected under a null model of random assemblage composition, whereas a decrease in their value (negative values) indicates phylogenetic clustering or close relatedness of taxa in the assemblage than those expected under a null model of random assemblage composition^6,69^.

### Climatic variables

Temperature and precipitation extremes have been widely recognised as the most important climatic factors in governing diversity and geographic distribution of groups of organisms eg.^45–47,70^. To describe the overall climate in the study area throughout the Holocene, we extracted annual mean temperature (bio1), annual precipitation (bio12), minimum temperature of coldest month (bio6, hereafter ‘temp_cold’), and precipitation of warmest quarter or summer precipitation (bio18, hereafter ‘prec_summer’), and precipitation of coldest quarter or winter precipitation (bio19, hereafter ‘prec_winter’) from the CHELSA-TraCE21k database^71^. CHELSA-TraCE21k takes account of orographic variation and provides monthly climate values for temperature and precipitation at 30 arcsecond (approximately 1 km) spatial resolution in 100-year time periods for the last 21,000 years. We used three climatic variables, namely ‘temp_cold’ (bio6), ‘prec_summer’ (bio18), and ‘prec_winter’ (bio19) as climatic predictor variables in our analyses. These variables represent extreme climates that may act as environmental filters in constraining taxon abundances and geographic distributions, and hence may govern phylogenetic affinity among co-occurring taxa over the Holocene^45–47,70^. We then predicted these climate indicator variables for each assemblage using natural splines.

### Numerical analyses

#### Data preparation

We averaged climatic variables, number of samples, sample age, age uncertainty, and PD metrics (sedMPD and sesMNTD) for every 1° latitude along the latitudinal range of our study area and for every 200 years in the Holocene (past 12,000 years). This data matrix thus has a spatial resolution of 1° latitude and a temporal resolution of 200 years. It was used to analyse the spatio-temporal patterns of these variables.

#### Latitudinal pattern in phylogenetic dispersion

To analyse the overall latitudinal patterns in PD during the Holocene, we fitted hierarchical generalised additive models (hGAM) using the framework of Pedersen et al*.*^72^, in which we treated ‘latitude’, ‘age’, ‘age’ grouped by ‘dataset_id’, and tensor product (interacton) of ‘latitude’ and ‘age’ as fixed-effect variables, and factorial ‘dataset_id’ as a random-effect variable. We treated ‘age uncertainty index’ of each sample as a weight in the model. We performed hGAM using the ‘mgcv’ package in R^73^, assuming a ‘gaussian’ distribution of errors (Supplementary text S1A).

#### Relationship between climate and phylogenetic dispersion

Estimates of PD as well as climate incorporate variation along latitude, longitude, and time. In addition to systematic variation, there may also be confounded random variation in climate as well as PD datasets from various sources—location-specific (variation among datasets for each latitude and longitude), time-specific (variation among samples of each dataset), and variable-specific (unique random for climate and PD estimates). Simple correlations between PD and climate may not be robust due to multi-dimensionality in both types of variables and complexity in their potential random variation. Therefore, we visualised the concordance between PD and climate indirectly. We first developed separate hGAMs for the spatio-temporal pattern of PD and for each climatic variables, while accounting for potential random variation. We then correlated the predicted values of hGAMs of PD and climate. We treated ‘latitude’ as a predictor variable, and ‘age, ‘age’ grouped by ‘dataset_id’ and interaction of ‘latitude’ and ‘age’ as fixed-effect variables, factorial ‘dataset_id’ as a random-effect variable, and ‘age-uncertainty index’ of each sample as a weight in the model (Supplementary text S1B). We then plotted the predicted values of hGAM of PD metrics against those of each climatic variable by fitting a simple ‘loess’ smoother. Statistical significance of these correlations is tested by a Procrustes randomization test (see below).

#### Temporal variation in latitudinal pattern of phylogenetic dispersion

To analyse temporal variation in the latitudinal patterns (spatio-temporal variation) of PD, we categorised the estimates of PD into time-periods (period) of 1000-years, based on sample-age. Hence, we have 12 periods through the Holocene. We then developed a hGAM, where PD was a response, ‘latitude’ and ‘age’ were fixed-effect variables each with a factor-smooth of ‘period’ assuming a separate (random) slope of the latitudinal pattern for each period, ‘dataset_id’ was a random-effect variable, and ‘age-uncertainty index’ of each sample was a weight in the model (Supplementary text S1C). This model is different from that for the Holocene-wide latitudinal pattern in that here we do not force the assumption of a single (global) smoother of latitude for the entire Holocene and allow for independent smoothing (wiggliness) and penalty of the latitudinal pattern of PD for each period. This allowed us to compare the smooths (slopes) of the models for each period against each other.

We followed the method of Rose et al*.*^74^ for comparing factor-smooths of each pair of periods. The difference in model smooths at a given point of latitude across all periods, results in a matrix of differences in smooths at all latitudes across all periods, here called a ‘difference matrix’. We plotted this matrix as diagrams, each with the difference in smooths of latitudinal pattern between a given pair of periods along the y-axis and latitude along the x-axis for all possible pairs of periods. A comparison pair in which there is a deviation of the 95% confidence interval of the smooth difference trendline (i.e., confidence interval does not include the zero-threshold and departs from the zero-threshold line by at least ± 0.05 through at least 2° continuous variation in latitude along the latitudinal gradient) is inferred as a significant difference between estimated smooths.

#### Climatic correlations with the spatio-temporal pattern of phylogenetic dispersion

To test the correlation of the spatio-temporal variation in PD with selected climatic variables, we created a difference matrix for each climatic variable at the same spatial (1° latitude) and temporal (1000-year period) resolution as in the difference matrix of spatio-temporal variation in PD. We standardised the difference matrices of PD and climatic variables using the ‘decostand’ function in the ‘vegan’ package^75^ and performed a Principal Component Analysis (PCA) of each matrix. We then performed a Procrustes randomisation test between the PCA of the difference matrix of the PD and that of the climatic difference matrix to test the degree of concordance between the spatio-temporal variation in PD and climate during the Holocene. In this method, ordination matrices are compared by rigid rotation, translation, reflection, and dilation to minimize the ‘sum of squares’ residual deviations between points for each observation. This provides a measure of the goodness-of-fit between the two ordinations or matrices. Statistical significance of this measure is assessed by randomisation tests^76^.

### Supplementary Information

Supplementary Information.

## Data Availability

The data and the R script used for analyses are available at https://github.com/HOPE-UIB-BIO/Latitudinal_phylodiversity_publication.
