# Supplementary material for: Latitudinal gradients in the phylogenetic assembly of angiosperms in Asia during the Holocene
Source: Sci Rep. 2024 Aug 2;14:17940. doi: 10.1038/s41598-024-67650-1 (PMC11297032; doi:10.1038/s41598-024-67650-1)
Supplement: Supplementary file 1 — Supplementary Information. [file 41598_2024_67650_MOESM1_ESM.docx]

**Supplementary material**

**Supplementary text S1. Hierarchical generalised additive models (hGAM) developed for the analysis of latitudinal patterns in phylogenetic dispersion (standardised effect size of mean pairwise phylogenetic distance (sesMPD) and standardised effect size of mean nearest taxon distance (sesMNTD)).**

**(A)** Overall (the Holocene-wide) latitudinal patterns in sesMPD and sesMNTD

*model <- mgcv::gam(y ~*

*s(latitude, k = 10, bs = "tp") +*

*s(age, k = 10, bs = "tp") +*

*s(age, by = dataset_id, bs = 'tp', m = 1) +*

*s(dataset_id, k = 99, bs = 're') +*

*ti(lat, age, bs = c("tp", "tp")),*

*weights = age_uncertainty_index,*

*method = "REML",*

*family = "gaussian”),*

where ‘y’ = response variable (sesMPD and sesMNTD), ‘age’ = calibrated age of the samples, ‘lat’ = latitude, ‘dataset_id’ = location.

**(B)** Climatic correlates of the spatio-temporal variation in the phylogenetic dispersion (sesMPD, sesMNTD) of angiosperm assemblages

*model <- mgcv::gam(y ~*

*s(latitude, k = 10, bs = 'tp') +*

*s(age, k = 10, bs = 'tp') +*

*s(age, by = dataset_id, bs = 'tp', m = 1) +*

*s(dataset_id, k = 99, bs = 're') +*

*ti(lat, age, bs = c('tp', 'tp')),*

*weights = age_uncertainty_index,*

*method = "REML",*

*family = "gaussian"),*

where ‘y’ = response variable (sesMPD, sesMNTD, and each of climatic variables), ‘age’ = calibrated age of the samples, ‘lat’ = latitude, ‘dataset_id’ = location.

**(C)** Temporal variation in latitudinal pattern of phylogenetic dispersion (sesMPD, sesMNTD) of angiosperm assemblages.

*model<- mgcv::gam(y ~*

*lat +*

*s(lat, by = period, bs = 'tp', m = 1 ) +*

*s(age, k = 10, bs = "tp") +*

*s(dataset_id, k = 99, bs = 're') +*

*s(period, k = 12, bs = 'fs') +*

*ti(lat, age, by = period, bs = c("tp", "tp")),*

*weights = age_uncertaintys_index, method = "REML",*

*family = "gaussian"),* where y is the response variable (sesMPD or sesMNTD).

**
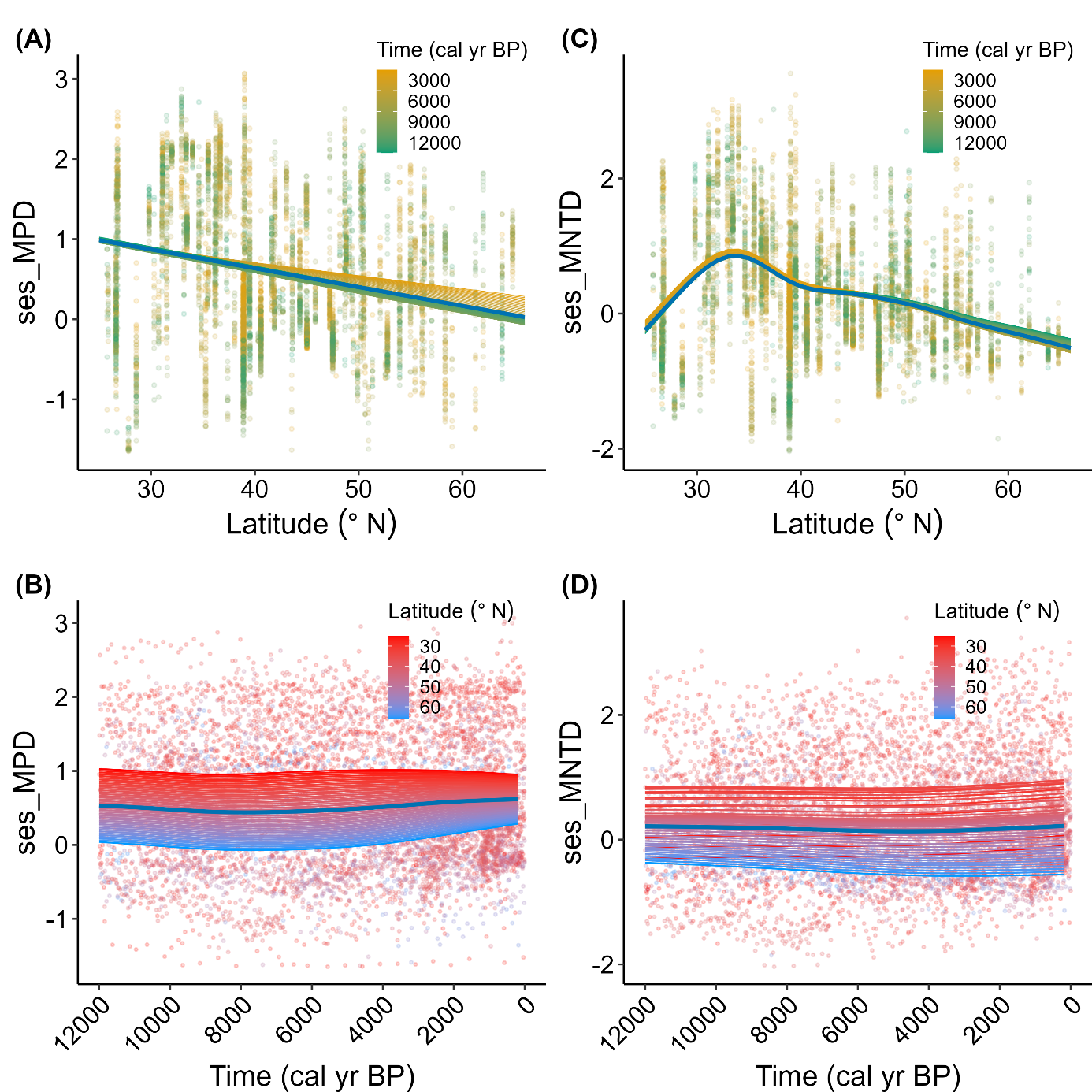
**

**Figure S1. Spatio-temporal pattern of phylogenetic dispersion – PD (standardised effect size of mean pairwise phylogenetic distance (sesMPD), standardised effect size of mean nearest taxon distance (sesMNTD)).** **(A)** The Holocene latitudinal pattern, and **(B)** spatio-temporal variation in sesMPD; and **(C)** the Holocene latitudinal pattern, and **(D)** spatio-temporal variation in sesMNTD of angiosperm families in central Asia. Each curve for both metrics represents variation in the metrics for every **(A, C)** 200-year time interval and **(B, D)** 1^o^ latitude. Dots in the background represent actual estimates of sesMPD and sesMNTD.

**
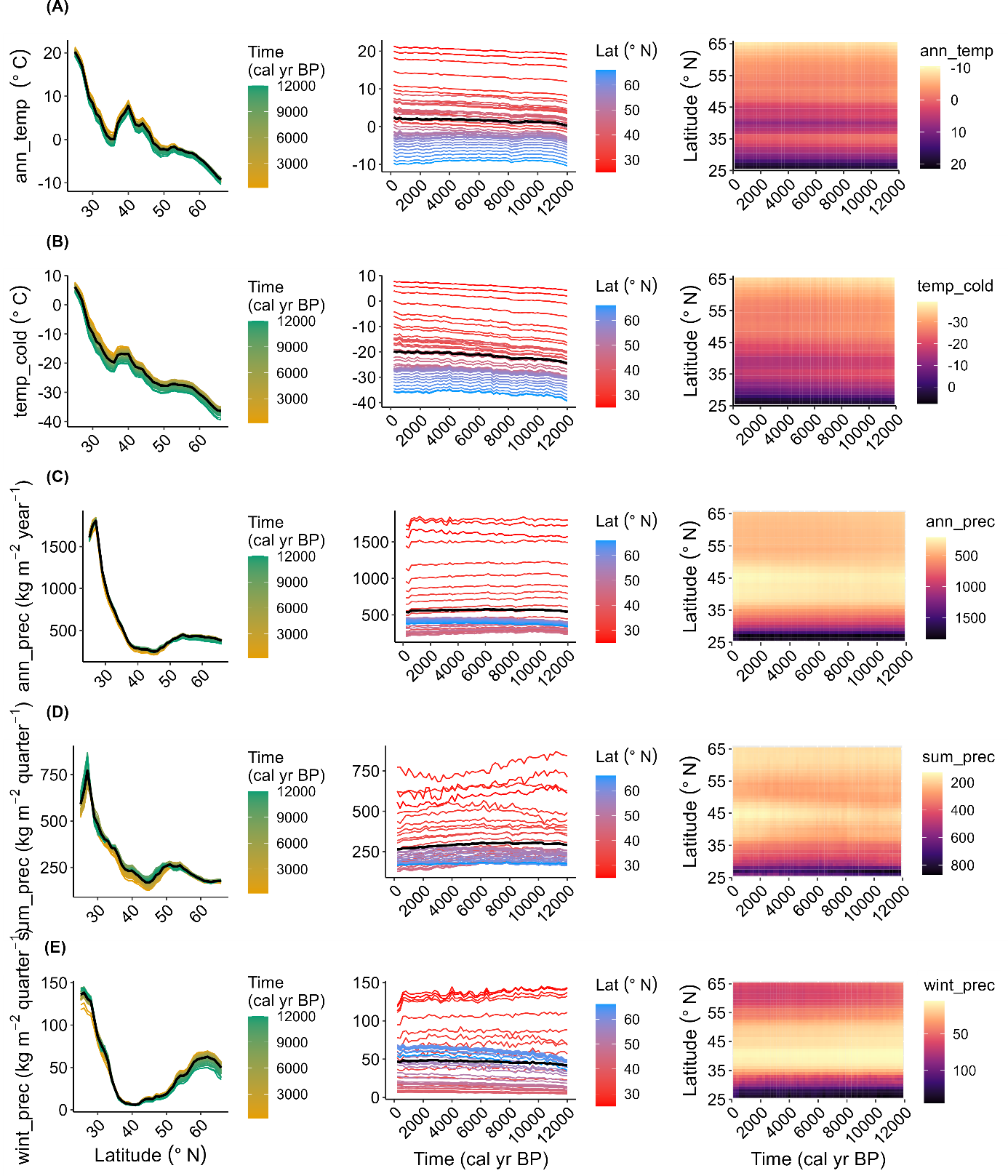
**

**Figure S2.** **Spatio-temporal patterns of climatic variables. (A)** annual mean temperature (ann_temp), **(B)** minimum temperature of the coldest month (temp_cold), **(C)** annual precipitation (ann_prec), **(D)** summer precipitation (sum_prec), and **(E)** winter precipitation (wint_prec) in the study area. In the first (left) column for each climatic variable, the gradient of colour represents the variation in the variable for every 200-years along the temporal gradient of 12000 years, where each line represents the latitudinal variation in the variable for every 200-years. The black line represents the overall (Holocene-wide or mean for 12000-years) latitudinal variation in the climatic variable. The middle column for each variable shows the temporal variation in the variable for every 1^o^ latitude along the latitudinal gradient, and the last (right) column for each variable shows the spatio-temporal variation in the variable for every 1^o^ variation in latitude and 200-year variation in time.

**
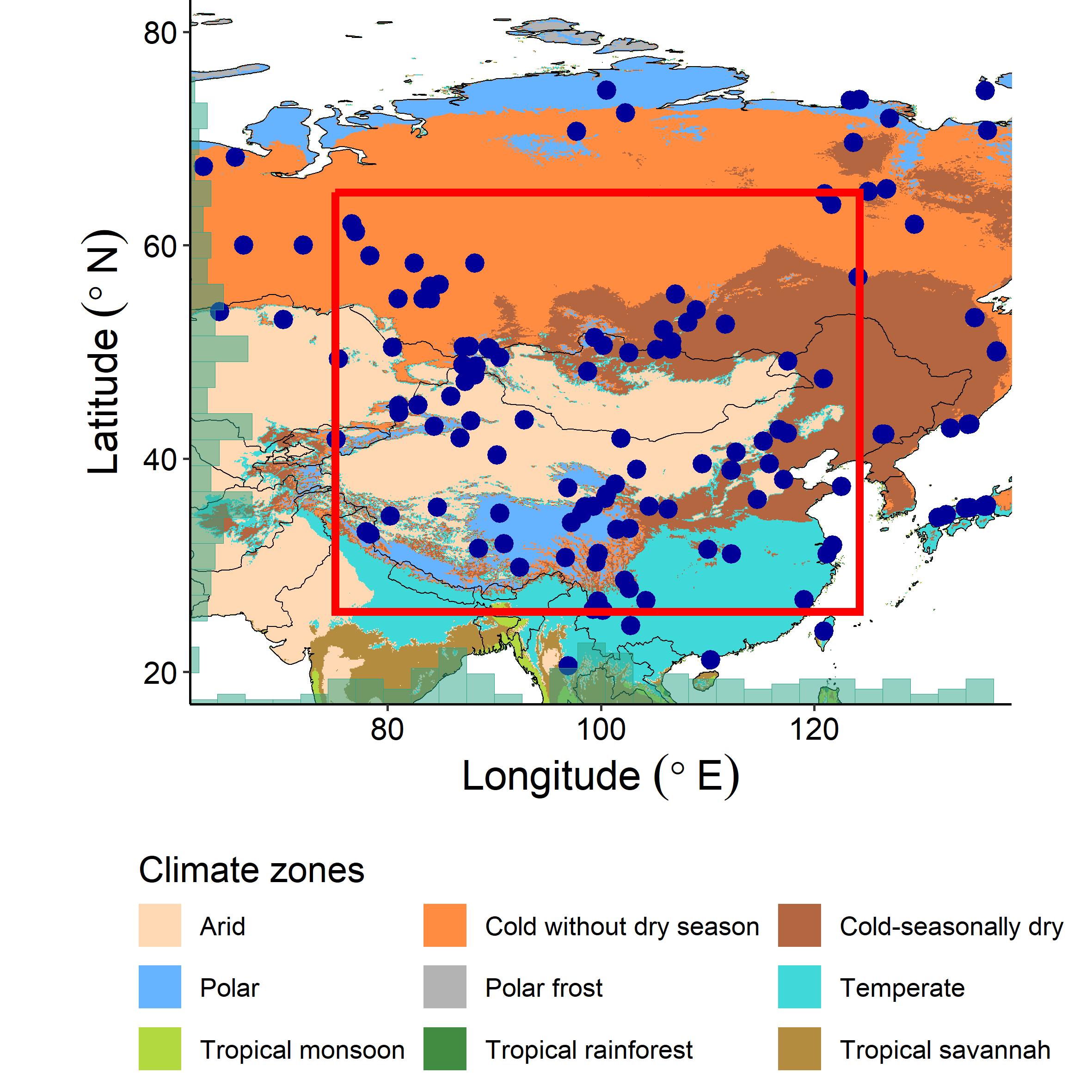
**

**Figure S3. Study area with the locations of the fossil pollen records.** Records within the red square were used for analysis. We used a raster from the geo-tiff file in Beck *et al.* ^1^ for the climate zonation and adopted their 13 climate-zone (general zones cluster) scheme. For simplicity of climatic zonation over space, we amalgamated the closely related climate-zones based on the five climate-zone scheme of Beck *et al.* ^1^. Hence, ‘Arid desert’ and ‘Arid steppe’ zones are merged into the ‘Arid’ zone, ‘Cold dry summer’ and ‘Cold dry winter’ zones are merged into the ‘Cold-seasonally dry’ zone, and ‘Temperate dry summer’, ‘Temperate dry winter’ and ‘Temperate without dry season’ zones are merged into the ‘Temperate’ zone.

**
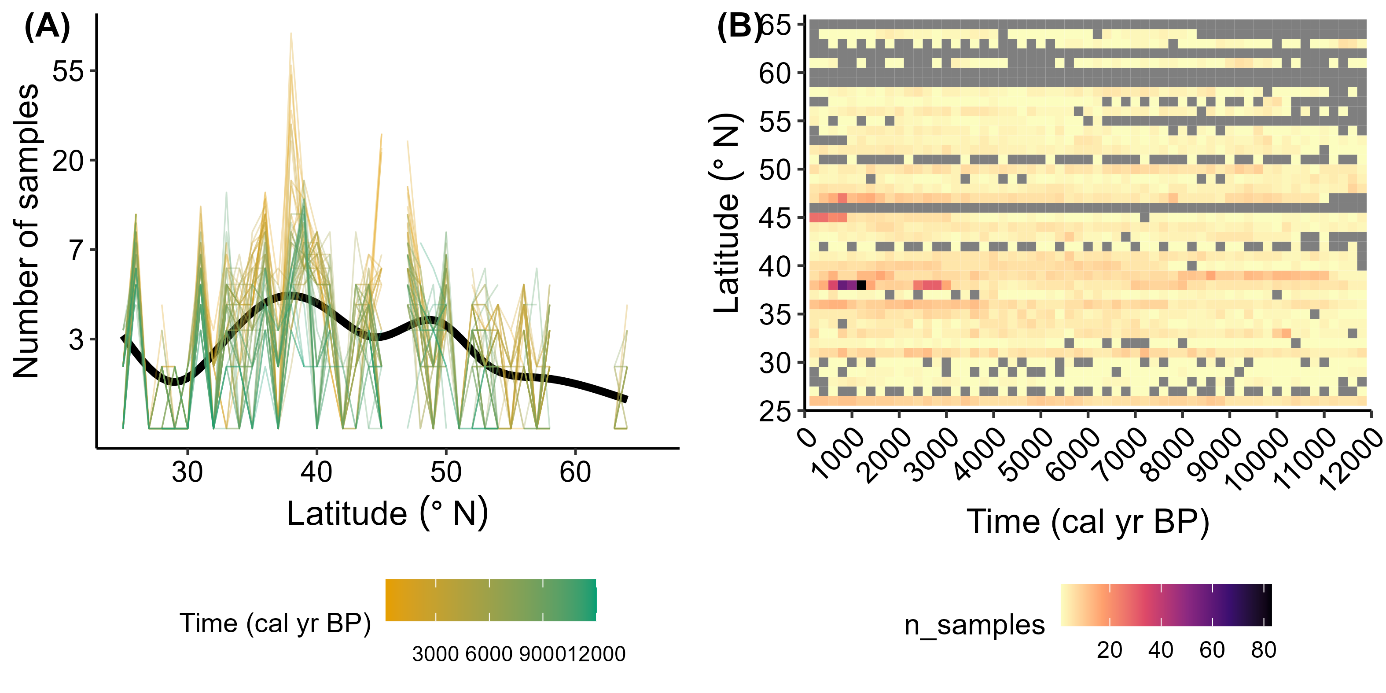
**

**Figure S4.** **Number of samples in the 99 fossil pollen records used along space and time.** **(A)** Number of samples along the latitudinal gradient. Gradient of colour represents the variation in the number of samples for every 200-years along the temporal gradient of 12000 years, where each line represents the log-transformed number of samples for each 200-years, and the y-axis labels show the actual number of samples. The thick black curve is fitted as a generalised additive model smoother, representing the overall latitudinal variation in the number of samples; **(B)** Spatio-temporal variation in the number of samples for every 1^o^ latitudinal band and 200-year variation in time along the temporal gradient of 12000 years. Grey cells represent cells lacking data.


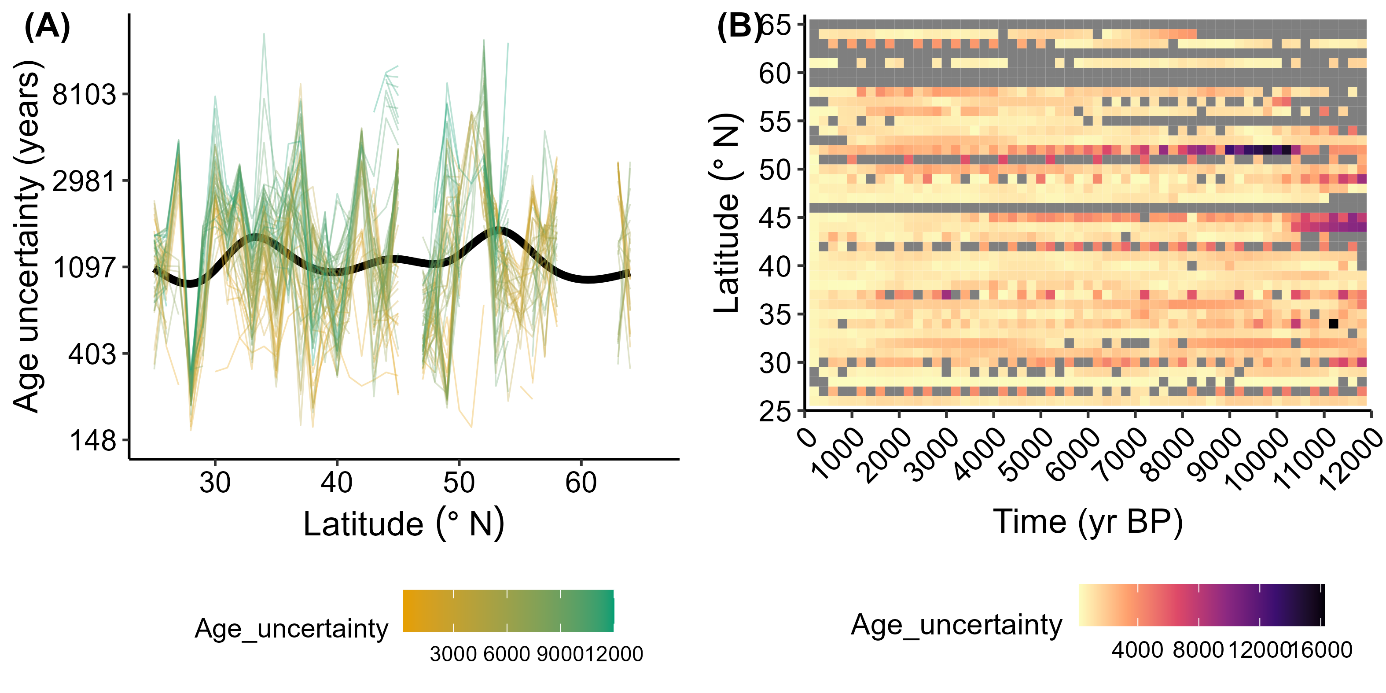


**Figure S5.** **Age-uncertainty (difference in the upper and lower quantiles of calibrated age) of the samples in the 99 fossil pollen records used in relation to space and time.** **(A)** age-uncertainty along the latitudinal gradient. Gradient of colour represents the variation in the age-uncertainty for every 200-year interval along the temporal gradient of 12000 years, where each line represents the log-transformed age-uncertainty for each 200-years, and the y-axis labels show actual age-uncertainty of the samples. The blue curve is fitted as a generalised additive model smoother, representing the overall latitudinal variation in the age-uncertainty of samples; **(B)** spatio-temporal variation in the age-uncertainty of samples for every 1^o^ latitudinal band and 200-year variation in time along the temporal gradient of 12000 years. Grey cells represent cells lacking data.

**Table S1.** **Statistical summary of hierarchical generalised additive models (hGAM) for the temporal variation in latitudinal pattern of standardised effect size of mean pairwise phylogenetic distance (sesMPD) and standardised effect size of mean nearest taxon distance (sesMNTD).** Here, sesMPD and sesMNTD are treated as response variables: latitude (lat), age, age grouped within period, and tensor product of latitude and age are fixed-effect variables; and location (factorial) and period (factorial), are treated as random-effect variables. Std error = standard error, Sig. codes = significance codes, ti = tensor product of the variables within parentheses, edf = estimated degrees of freedom, Ref. df = reference degrees of freedom used in computing test statistic and p-values.

| Metric | Component | | Term | | Estimate | | Std Error | | | t-value | | p-value | |  | |
| --- | --- | --- | --- | --- | --- | --- | --- | --- | --- | --- | --- | --- | --- | --- | --- |
| ses  MPD | Parametric coefficients | | (Intercept) | | 2.01 | | 0.38 | | | 5.28 | | < 0.001 | | *** | |
|  |  |  | Lat | | -0.03 | | 0.01 | | | -3.88 | | < 0.001 | | *** | |
|  | **Component** | | **Term** | | **Edf** | | **Ref. df** | | | **F-value** | | **p-value** | |  | |
|  | Smooth terms | | s(lat):period1000 | | 6.09 | | 8.00 | | | 171.39 | | < 0.001 | | *** | |
|  |  |  | s(lat):period2000 | | 6.38 | | 8.00 | | | 72.92 | | < 0.001 | | *** | |
|  |  |  | s(lat):period3000 | | 0.00 | | 8.00 | | | 0.00 | | 0.268 | |  | |
|  |  |  | s(lat):period4000 | | 0.00 | | 8.00 | | | 0.00 | | 0.340 | |  | |
|  |  |  | s(lat):period5000 | | 0.00 | | 8.00 | | | 0.00 | | 0.336 | |  | |
|  |  |  | s(lat):period6000 | | 6.78 | | 8.00 | | | 11.18 | | < 0.001 | | *** | |
|  |  |  | s(lat):period7000 | | 5.88 | | 8.00 | | | 15.37 | | < 0.001 | | *** | |
|  |  |  | s(lat):period8000 | | 7.01 | | 8.00 | | | 39.83 | | < 0.001 | | *** | |
|  |  |  | s(lat):period9000 | | 3.76 | | 8.00 | | | 2.91 | | 0.005 | | ** | |
|  |  |  | s(lat):period10000 | | 7.08 | | 8.00 | | | 64.26 | | < 0.001 | | *** | |
|  |  |  | s(lat):period11000 | | 4.17 | | 8.00 | | | 4.53 | | 0.003 | | ** | |
|  |  |  | s(lat):period12000 | | 2.80 | | 8.00 | | | 1.93 | | 0.083 | | . | |
|  |  |  | s(age) | | 6.30 | | 7.32 | | | 31.66 | | < 0.001 | | *** | |
|  |  |  | s(age):period1000 | | 0.00 | | 6.00 | | | 0.00 | | 0.725 | |  | |
|  |  |  | s(age):period2000 | | 0.00 | | 6.00 | | | 0.00 | | 0.738 | |  | |
|  |  |  | s(age):period3000 | | 0.51 | | 6.00 | | | 0.19 | | 0.121 | |  | |
|  |  |  | s(age):period4000 | | 0.00 | | 7.00 | | | 0.00 | | 0.511 | |  | |
|  |  |  | s(age):period5000 | | 0.00 | | 6.00 | | | 0.00 | | 0.384 | |  | |
|  |  |  | s(age):period6000 | | 0.89 | | 7.00 | | | 0.50 | | 0.037 | | * | |
|  |  |  | s(age):period7000 | | 0.00 | | 6.00 | | | 0.00 | | 0.697 | |  | |
|  |  |  | s(age):period8000 | | 0.00 | | 6.00 | | | 0.00 | | 0.875 | |  | |
|  |  |  | s(age):period9000 | | 2.71 | | 7.00 | | | 2.92 | | < 0.001 | | *** | |
|  |  |  | s(age):period10000 | | 0.00 | | 6.00 | | | 0.00 | | 0.659 | |  | |
|  |  |  | s(age):period11000 | | 0.00 | | 6.00 | | | 0.00 | | 0.298 | |  | |
|  |  |  | s(age):period12000 | | 0.67 | | 5.00 | | | 0.47 | | 0.045 | | * | |
|  |  |  | s(dataset_id) | | 94.78 | | 97.00 | | | 159.77 | | < 0.001 | | *** | |
|  |  |  | s(period) | | 0.00 | | 11.00 | | | 0.00 | | 0.750 | |  | |
|  |  |  | ti(lat,age):  period1000 | | 4.85 | | 5.26 | | | 4.2 | | < 0.001 | | *** | |
|  |  |  | ti(lat,age):  period2000 | | 1.00 | | 1.00 | | | 0.66 | | 0.416 | |  | |
|  |  |  | ti(lat,age):  period3000 | | 2.34 | | 2.90 | | | 1.60 | | 0.151 | |  | |
|  |  |  | ti(lat,age):  period4000 | | 5.08 | | 6.30 | | | 2.95 | | 0.009 | | * | |
|  |  |  | ti(lat,age):  period5000 | | 1.00 | | 1.00 | | | 1.04 | | 0.305 | |  | |
|  |  |  | ti(lat,age):  period6000 | | 1.71 | | 2.10 | | | 0.98 | | 0.333 | |  | |
|  |  |  | ti(lat,age):  period7000 | | 1.00 | | 1.00 | | | 9.90 | | 0.001 | | ** | |
|  |  |  | ti(lat,age):  period8000 | | 1.00 | | 1.00 | | | 0.25 | | 0.614 | |  | |
|  |  |  | ti(lat,age):  period9000 | | 5.80 | | 6.55 | | | 3.35 | | 0.001 | | ** | |
|  |  |  | ti(lat,age):  period10000 | | 1.00 | | 1.00 | | | 0.10 | | 0.742 | |  | |
|  |  |  | ti(lat,age):  period11000 | | 1.00 | | 1.00 | | | 4.00 | | 0.045 | | * | |
|  |  |  | ti(lat,age):  period12000 | | 1.00 | | 1.00 | | | 1.49 | | 0.221 | |  | |
| Sig. codes: '***' < 0.001 < '**' < 0.01 < '*' < 0.05 < '.' < 0.1 < ' ' < 1; adjusted R-squared: 0.759; deviance explained 0.768; N = 6557 | | | | | | | | | | | | | | |  |
| Metric | | **Component** | | **Term** | | **Estimate** | | **Std Error** | **t-value** | | **p-value** | |  | |  |
| ses  MNTD | | Parametric coefficients | | (Intercept) | | 1.39 | | 0.35 | 3.92 | | < 0.001 | | *** | |  |
|  |  |  |  | lat | | -0.02 | | 0.01 | -3.22 | | 0.001 | | ** | |  |
|  |  | **Component** | | **Term** | | **edf** | | **Ref. df** | **F-value** | | **p-value** | |  | |  |
|  |  | Smooth terms | | s(lat):period1000 | | 6.47 | | 8.00 | 311.90 | | < 0.001 | | *** | |  |
|  |  |  |  | s(lat):period2000 | | 6.54 | | 8.00 | 91.51 | | < 0.001 | | *** | |  |
|  |  |  |  | s(lat):period3000 | | 5.82 | | 8.00 | 11.23 | | < 0.001 | | *** | |  |
|  |  |  |  | s(lat):period4000 | | 4.92 | | 8.00 | 5.04 | | < 0.001 | | *** | |  |
|  |  |  |  | s(lat):period5000 | | 2.59 | | 8.00 | 1.13 | | 0.031 | | * | |  |
|  |  |  |  | s(lat):period6000 | | 0.00 | | 8.00 | 0.00 | | 0.537 | |  | |  |
|  |  |  |  | s(lat):period7000 | | 3.74 | | 8.00 | 2.79 | | 0.010 | | * | |  |
|  |  |  |  | s(lat):period8000 | | 6.24 | | 8.00 | 18.58 | | < 0.001 | | *** | |  |
|  |  |  |  | s(lat):period9000 | | 2.26 | | 8.00 | 1.00 | | 0.008 | | ** | |  |
|  |  |  |  | s(lat):period10000 | | 5.23 | | 8.00 | 9.62 | | < 0.001 | | *** | |  |
|  |  |  |  | s(lat):period11000 | | 4.38 | | 8.00 | 4.97 | | < 0.001 | | *** | |  |
|  |  |  |  | s(lat):period12000 | | 5.92 | | 8.00 | 18.15 | | < 0.001 | | *** | |  |
|  |  |  |  | s(age) | | 1.53 | | 1.81 | 11.38 | | 0.001 | | ** | |  |
|  |  |  |  | s(age):period1000 | | 1.48 | | 6.00 | 2.90 | | < 0.001 | | *** | |  |
|  |  |  |  | s(age):period2000 | | 1.59 | | 7.00 | 2.32 | | < 0.001 | | *** | |  |
|  |  |  |  | s(age):period3000 | | 1.95 | | 7.00 | 2.30 | | < 0.001 | | *** | |  |
|  |  |  |  | s(age):period4000 | | 0.00 | | 7.00 | 0.00 | | 0.55 | |  | |  |
|  |  |  |  | s(age):period5000 | | 0.86 | | 6.00 | 0.58 | | 0.049 | | . | |  |
|  |  |  |  | s(age):period6000 | | 0.00 | | 6.00 | 0.00 | | 0.834 | |  | |  |
|  |  |  |  | s(age):period7000 | | 0.00 | | 6.00 | 0.00 | | 0.953 | |  | |  |
|  |  |  |  | s(age):period8000 | | 0.89 | | 6.00 | 0.64 | | 0.057 | | . | |  |
|  |  |  |  | s(age):period9000 | | 0.00 | | 6.00 | 0.00 | | 0.462 | |  | |  |
|  |  |  |  | s(age):  period10000 | | 0.00 | | 6.00 | 0.00 | | 0.957 | |  | |  |
|  |  |  |  | s(age):  period11000 | | 0.77 | | 6.00 | 0.61 | | 0.043 | | * | |  |
|  |  |  |  | s(age):  period12000 | | 0.00 | | 5.00 | 0.00 | | 0.324 | |  | |  |
|  |  |  |  | s(dataset_id) | | 94.20 | | 97.00 | 130.81 | | < 0.001 | | *** | |  |
|  |  |  |  | s(period) | | 0.00 | | 11.00 | 0.00 | | 0.856 | |  | |  |
|  |  |  |  | ti(lat,age):  period1000 | | 4.16 | | 4.68 | 1.26 | | 0.306 | |  | |  |
|  |  |  |  | ti(lat,age):  period2000 | | 2.84 | | 3.33 | 0.05 | | 0.987 | |  | |  |
|  |  |  |  | ti(lat,age):  period3000 | | 2.08 | | 2.29 | 0.62 | | 0.456 | |  | |  |
|  |  |  |  | ti(lat,age):  period4000 | | 2.31 | | 2.55 | 1.58 | | 0.152 | |  | |  |
|  |  |  |  | ti(lat,age):  period5000 | | 1.00 | | 1.00 | 0.06 | | 0.795 | |  | |  |
|  |  |  |  | ti(lat,age):period6000 | | 2.11 | | 2.37 | 2.41 | | 0.069 | | . | |  |
|  |  |  |  | ti(lat,age):  period7000 | | 1.50 | | 1.64 | 1.42 | | 0.331 | |  | |  |
|  |  |  |  | ti(lat,age):  period8000 | | 2.65 | | 3.27 | 0.08 | | 0.977 | |  | |  |
|  |  |  |  | ti(lat,age):  period9000 | | 3.96 | | 4.77 | 1.70 | | 0.080 | | . | |  |
|  |  |  |  | ti(lat,age):  period10000 | | 1.66 | | 1.84 | 0.86 | | 0.312 | |  | |  |
|  |  |  |  | ti(lat,age):  period11000 | | 1.00 | | 1.00 | 0.20 | | 0.647 | |  | |  |
|  |  |  |  | ti(lat,age):  period12000 | | 1.00 | | 1.00 | 0.23 | | 0.630 | |  | |  |
| Sig. codes: 0 <= '***' < 0.001 < '**' < 0.01 < '*' < 0.05 < '.' < 0.1 < ' ' < 1; adjusted R-squared: 0.745; deviance explained 0.753, N = 6557 | | | | | | | | | | | | | | |  |

**References**

1. Beck, H. E. *et al.* Present and future Köppen-Geiger climate classification maps at 1-km resolution. *Sci. Data* **5**, 1–12 (2018).
